# Effective Synthesis and Antifouling Activity of Dolastatin 16 Derivatives

**DOI:** 10.3390/md20020124

**Published:** 2022-02-04

**Authors:** Loida O. Casalme, Keisuke Katayama, Yoshiki Hayakawa, Kensuke Nakamura, Arisa Yamauchi, Yasuyuki Nogata, Erina Yoshimura, Fuyuhiko Matsuda, Taiki Umezawa

**Affiliations:** 1 Division of Environmental Materials Science, Graduate School of Environmental Science, Hokkaido University, Sapporo 060-0810, Japan; locasalme@ees.hokudai.ac.jp (L.O.C.); kkata@ees.hokudai.ac.jp (K.K.); e76173132@ees.hokudai.ac.jp (Y.H.); c32538at@ees.hokudai.ac.jp (K.N.); 1210910002e@ees.hokudai.ac.jp (A.Y.); fmatsuda@ees.hokudai.ac.jp (F.M.); 2 Sustainable System Research Laboratory, Central Research Institute of Electric Power Industry, Abiko 270-1194, Japan; noga@criepi.denken.or.jp; 3 CERES, Inc., Abiko 270-1153, Japan; yoshimura@ceresco.jp

**Keywords:** natural product, antifouling, dolastatin 16, peptide, *Amphibalanus amphitrite*

## Abstract

Some derivatives of dolastatin 16, a depsipeptide natural product first obtained from the sea hare *Dolabella auricularia*, were synthesized through second-generation synthesis of two unusual amino acids, dolaphenvaline and dolamethylleuine. The second-generation synthesis enabled derivatizations such as functionalization of the aromatic ring in dolaphenvaline. The derivatives of fragments and whole structures were evaluated for antifouling activity against the cypris larvae of *Amphibalanus amphitrite.* Small fragments inhibited the settlement of the cypris larvae at potent to moderate concentrations (EC_50_ = 0.60-4.62 μg/mL), although dolastatin 16 with a substituent on the aromatic ring (**24**) was much less potent than dolastatin 16.

## 1. Introduction

**Figure 1:**
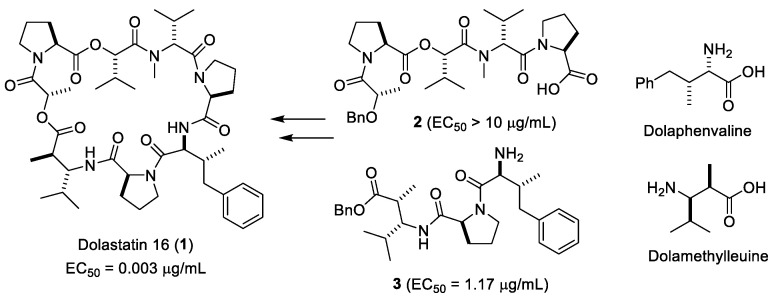
Unusual amino acids in dolastatin 16 and previous total synthesis.

Dolastatin 16 (**1**, Figure 1), a depsipeptide natural product obtained from the sea hare *Dolabella auricularia*, was first reported by Pettit and co-workers in 1997 [1], and includes two unusual amino acids, dolaphenvaline and dolamethylleuine (Figure 1). Absolute configurations of the two amino acids were determined through X-ray crystallographic analysis in 2011 [2]. Pettit’s group showed strong growth inhibitions of human cancer cell lines such as NCI-H460, KM20L2 and SF-295 with **1** isolated from the natural sample. The same group also synthesized **1**, and evaluated the anticancer activities of synthetic **1** to reveal it to be much less potent than the isolated compound [3].

Highly powerful antifouling activity of **1** toward the larval settlement and metamorphosis of the barnacle *Amphibalanus amphitrite* was shown by Tan and co-workers in 2010 [4]. EC_50_ (50% effective concentration) and LC_50_ (50% lethal concentration) values of **1** are 0.003 and 20 μg/mL, respectively, suggesting it to be a promising lead compound for the development of a novel antifouling material. Organotin compounds such as tributyltin (TBT) or triphenyltin (TPT) were employed as antifouling compounds, resulting in serious pollution of the ocean environment [5,6,7,8,9,10,11,12,13,14]. Due to these negative influences, the use of organotin compounds was prohibited by the International Maritime Organization (IMO) in 2008 [15]. Alternative antifouling compounds, sea-nine 211 or copper pyrithione, have also been revealed to exert harmful influence on the ocean environment [16,17]. Thus, green alternative antifouling materials must be found to preserve the ocean environment. Toward this goal, many academic researchers have reported new antifouling compounds derived from natural products [18,19,20,21,22,23,24,25,26,27,28,29,30]. We have also engaged in the study of the antifouling natural products, 10-isocyano-4-cadinene [31,32,33], omaezallene [34,35,36] and dolastatin 16 (**1**) [37,38].

In 2017, we reported the total synthesis and antifouling activity of **1** and two intermediates, northern carboxylic acid fragment **2** and southern amine fragment **3**, to reveal highly potent activity of **1** (EC_50_ < 0.03 μg/mL) and moderate to low activities of **2** and **3** (EC_50_ > 10 and 1.17 μg/mL, respectively) [38]. With these results in hand, we envisioned that additional compounds related to **1** would show further potential toward the development of a green antifouling material. For quick access to these compounds, second-generation synthesis of the two unusual amino acids was required because derivatization of the amino acids was difficult with the previous methodology [37]. In this paper, we describe our efforts to synthesize derivatives of dolaphenvaline and dolamethylleuine as well as some derivatives of **1**. Evaluations of the antifouling activity toward the cypris larvae of the barnacle *A. amphitrite* were also conducted.

## 2. Results and Discussions

**Scheme 1:**
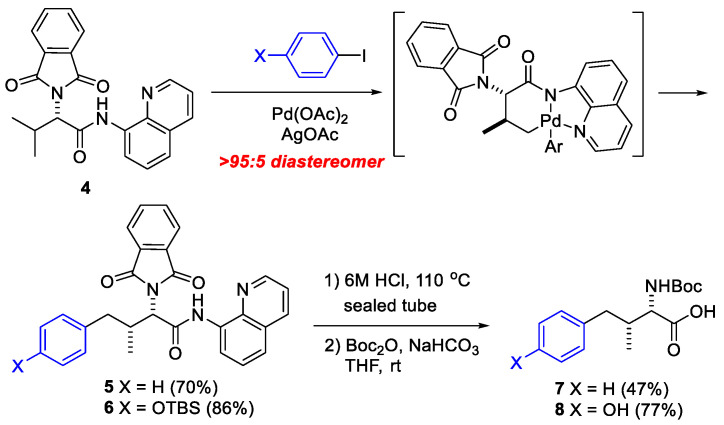
Syntheses of dolaphenvaline derivatives.

For the preparation of dolaphenvaline derivatives, the C-H activation reaction focused on amide **4**, which was obtained from l-valine in 3 steps according to known method [39]. The synthetic details to obtain dolaphenvaline derivatives **7** and **8** are shown in Scheme 1. The installment of the aromatic ring on **4** was accomplished in a regio- and diastereoselective manner, confirmed by ^1^H NMR, in the presence of a palladium catalyst and silver salt without solvent to give amide **5** [39] (see Supplementary Material Figures S1–S14 for NMR spectra). This high diastereoselectivity was rationalized by steric repulsion between the methyl group and the phthaloyl group in the transition state shown in the brackets. Acidic hydrolysis, followed by protection with a Boc group, afforded Boc-dolaphenvaline **7**, previously reported by us [37]. First synthesis of **8**, having a *p*-hydroxy group on the benzene ring, was also possible through the same pathway when *p*-siloxyiodobenzene was employed in the C-H activation step.

**Scheme 2:**
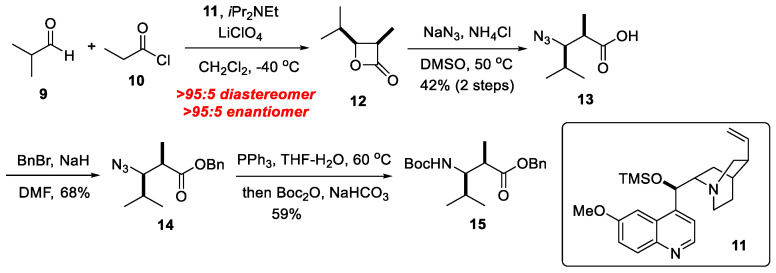
Synthesis of dolamethylleuine derivatives.

Boc-dolamethylleuine **15** was accessed through a [2+2] addition reaction in the presence of organocatalyst **11** as the key step to construct two contiguous asymmetric carbon centers, as shown in Scheme 2 [40]. The reaction between isovaleraldehyde (**9**) and propionyl chloride (**10**) at −40 °C provided volatile lactone **12** in a highly stereoselective manner (minor diastereomer could not be observed in crude ^1^H NMR), which was next converted into carboxylic acid **13** by the treatment with NaN_3_ and NH_4_Cl in DMSO. Before the Staudinger reaction, i.e., the conversion of the azide group to an amino group, benzyl ester formation was necessary since the Staudinger reaction of **13** resulted in low yield (<20%). The Staudinger reaction with benzyl ester **14** and subsequent protection with Boc_2_O proceeded smoothly to give **15** in 59% yield, previously reported by us (specific rotation of the current compound was completely identical with that of the previous one).

**Scheme 3:**
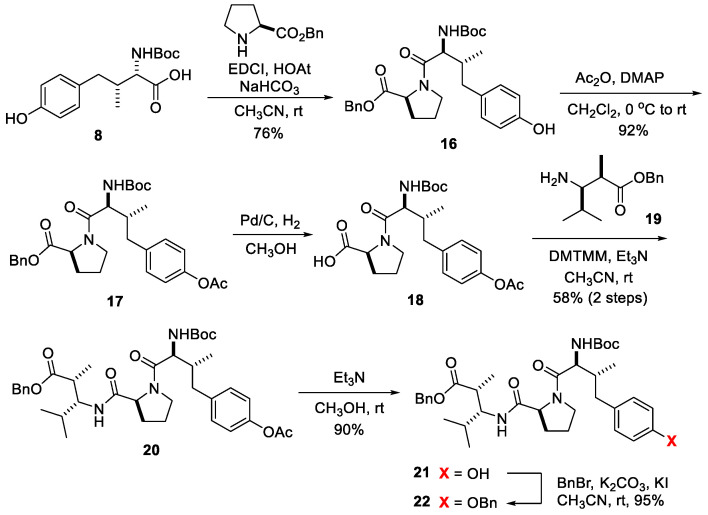
Synthesis of southern fragments. EDCI, 1-ethyl-3-(3-dimethylaminopropyl)carbodiimide hydrochloride; DMAP, 4-dimethylaminopyridine; DMTMM, 4-(4,6-dimethoxy-1,3,5-triazin-2-yl)-4-methylmorpholinium tetrafluoroborate.

With the effective synthetic route to the two unusual amino acids established, we launched the preparation of dolastatin 16 derivatives according to the previous report [38]. Condensation between **8** and proline benzyl ester gave amide **16** in which the hydroxy group was then acetylated under standard conditions (Scheme 3). After hydrogenolysis of peptide **17**, coupling of resulting **18** with dolamethylleuine benzyl ester **19** gave peptide **20**. In order to proceed with structure-activity relationship studies, **20** was converted into benzyl ether **22** in two steps through methanolysis of the acetate, followed by treatment of the resultant peptide **21** with BnBr, K_2_CO_3_ and KI.

**Scheme 4:**
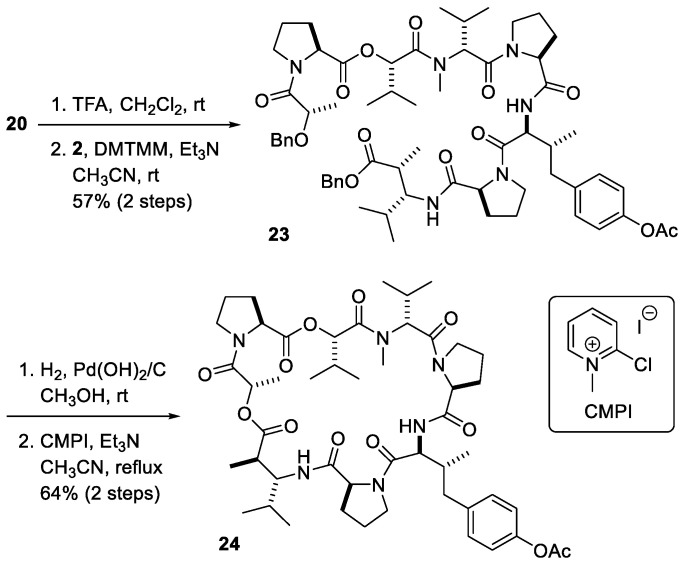
Synthesis of dolastatin 16 derivative. DMTMM, 4-(4,6-dimethoxy-1,3,5-triazin-2-yl)-4-methylmorpholinium tetrafluoroborate; CMPI, 2-chloro-1-methylpyridimium iodide.

Functionalized southern fragment **20** was further coupled with the northern fragment **2** to give peptide **23** for the macrolactonization reaction (Scheme 4). In the previous studies by Pettit and us, Shiina’s conditions by 2-methyl-6-nitrobenzoic anhydride (MNBA) [41,42,43] provided a low yield of **1** (22% by Pettit, 31% by us). In order to improve the reaction yield, extensive optimizations were performed, eventually finding that Mukaiyama’s conditions using 2-chloro-1-methylpyridimium iodide (CMPI) [44] gave the target compound **24** in 64% yield over 2 steps (deprotection of benzyl groups and macrolactonization).

**Scheme 5:**
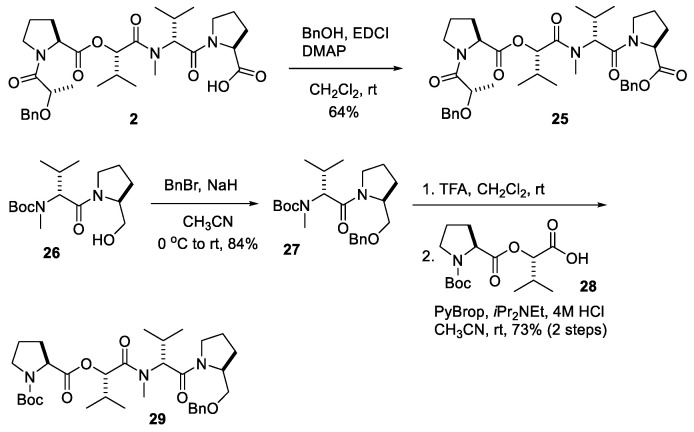
Synthesis of northern fragments. EDCI, 1-ethyl-3-(3-dimethylaminopropyl)carbodiimide hydrochloride; DMAP, 4-dimethylaminopyridine; PyBrop: bromotripyrrolidinophosphonium hexafluorophosphate.

Additional syntheses of northern fragments, benzyl ester **25** and benzyl ether **29**, were carried out as shown in Scheme 5. Benzyl ester **25** was prepared by esterification reaction of **2** in the presence of 1-ethyl-3-(3-dimethylaminopropyl)carbodiimide hydrochloride (EDCI). For the synthesis of **29**, installation of a benzyl group to the prolinol moiety at the stage of **26** was essential because direct etherification into **29** from the corresponding alcohol resulted in a complex mixture. The subsequent condensation reaction between carboxylic acid **28** and the amine obtained by removal of the Boc group of **27** [45] proceeded cleanly to give **29** in good yield.

**Figure 2:**
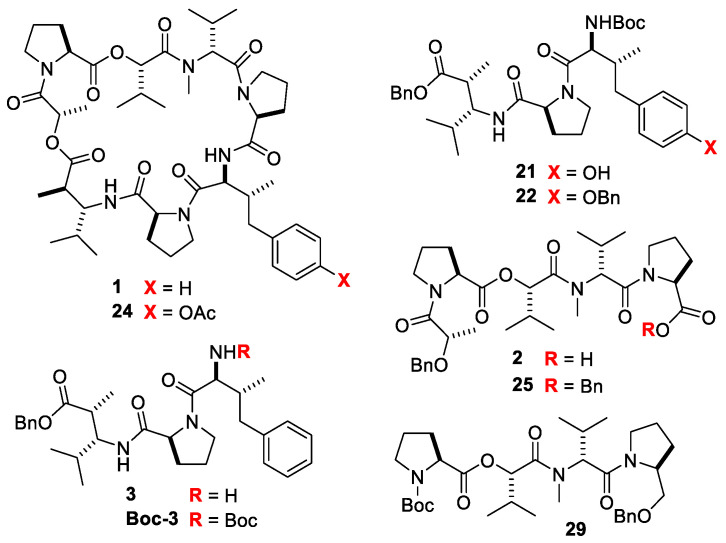
Compounds for evaluation of antifouling activity.

The antifouling activities of synthetic samples were evaluated as EC_50_ values against the cypris larvae of *A. amphitrite* by exposure of each compound for 48 h (Table 1, Figure 2). For comparison, EC_50_ values for the previous compounds **1**–**3** are also shown in the table. Installation of a functional group on the aromatic ring of **1** decreased the antifouling activity to moderate (**24**, EC_50_ = 1.74 μg/mL). We next investigated the biological activity of the fragments. All samples showed antifouling profiles with low toxicity against cypris larvae of the barnacle *A. amphitrite*. Among the fragments examined, compounds **Boc-3**, **21**, and **25** were more active with EC_50_ values below 1 μg/mL. Protection of the southern fragment with a Boc group improved the EC_50_ value (**Boc-3**, EC_50_ = 0.79 μg/mL). We believe this improvement is due its lower polarity than **3** (EC_50_ = 1.17 μg/mL) by protection of the amino group. It was revealed that functional groups at the *p*-position of the aromatic ring affected the antifouling activity of the southern fragment: a hydroxy group (**21**, EC_50_ = 0.60 μg/mL) had a slightly decreased EC_50_ value compared to **Boc-3**, but a benzyloxy group (**22**, EC_50_ = 4.62 μg/mL) dramatically reduced the antifouling activities to 4.62 μg/mL. These results indicate that steric bulkiness at this position affected the activity. A benzyl ester of the northern fragment (**25**, EC_50_ = 0.90 μg/mL) showed much higher potency than **2** (EC_50_ > 10 μg/mL). Again, the less polar fragment was more active than the corresponding more polar one. Interestingly, a benzyl ether (**29**, EC_50_ = 3.27 μg/mL) weakened the antifouling activity, suggesting the importance of the lactate moiety or the presence of a carbonyl group for the northern fragment.

## 3. Materials and Methods

### 3.1. General Methods

The IR spectra were recorded on a JASCO FTIR-4100 Type A spectrometer (JASCO corporation, Tokyo, Japan) using a NaCl cell. The ^1^H NMR and ^13^C NMR spectra were recorded using a JNM-EX 400 (400 MHz and 100 MHz) spectrometer (JEOL Ltd., Tokyo, Japan). Chemical shifts were reported in ppm relative to CHCl_3_ in CDCl_3_ for ^1^H NMR (δ = 7.26) and ^13^C NMR (δ = 77.0) and CHD_2_OH in CD_3_OD for ^1^H NMR (δ = 3.35) and ^13^C NMR (δ = 49.3). Splitting patterns for ^1^H NMR were designated as “s, d, t, q, m, dt, dd, and td”. These symbols indicate “singlet, doublet, triplet, quartet, multiplet, doublettriplet, doubletdoublet, and tripletdoublet”, respectively. All commercially obtained reagents were employed as received. Analytical TLC was carried out using pre-coated silica gel plates (Wako TLC Silicagel 70F_254_, FUJIFILM Wako Pure Chemical Coporation, Osaka, Japan). Wakogel 60N 63-212 μm was used for column chromatography.

### 3.2. Pht-Dpv-NH(8-quinoline) ***5***

Amide **4** (1.00 g, 2.67 mmol), AgOAc (891 mg, 5.36 mmol), Pd(OAc)_2_ (118 mg, 0.540 mmol) and iodobenzene (0.600 mL, 5.36 mmol) were added to a flask. The mixture was stirred for 6 h at 90 °C under Ar atmosphere. After being cooled to room temperature, the reaction was diluted with AcOEt and then filtered through a pad of celite wash with AcOEt and concentrated in vacuo. The residue was purified by silica gel column chromatography (Hexane:EtOAc = 80:20 then 70:30) to afford **5** (840 mg, 70%) as a white solid: ${\left[\alpha \right]}_{D}^{25}$ −36.1 (*c* 0.18, CHCl_3_); IR (neat) 3650, 2924, 1717, 1530, 1487, 1384, 1327, 1261, 1070, 792, 721 cm^–1^; ^1^H NMR (400 MHz, CDCl_3_) δ 0.87 (3H, d, *J* = 6.8 Hz), 2.43 (1H, dd, *J* = 13.4, 10.5 Hz), 3.23 (1H, dd, *J* = 13.7, 3.4 Hz), 3.36–3.40 (1H, m), 4.88 (1H, d, *J* = 10.7 Hz), 7.16–7.31 (5H, m), 7.46 (1H, dd, *J* = 8.3, 4.4 Hz), 7.53 (2H, d, *J* = 3.4 Hz), 7.74 (2H, dd, *J* = 5.4, 2.9 Hz), 7.88 (2H, dd, *J* = 5.4, 2.9 Hz), 8.16 (1H, dd, *J* = 8.3, 2.0 Hz), 8.77 (1H, t, *J* = 4.4 Hz), 8.86 (1H, dd, *J* = 4.4, 2.0 Hz), 10.68 (1H, brs); ^13^C NMR (CDCl_3_, 100 MHz) δ 15.9, 34.6, 40.8, 61.7, 117.3, 121.9, 122.3, 124.0, 126.4, 127.5, 128.6, 129.7, 131.8, 134.1, 134.5, 136.5, 139.0, 139.8, 148.8, 166.7, 168.3; HRMS (ESI) *m*/*z*:[M + Na]^+^; Calcd for C_28_H_23_N_3_O_3_Na 472.1631; Found 472.1632.

### 3.3. Boc-Dpv-OH ***7***

Amide **5** (148 mg, 0.330 mmol) was dissolved in 3.0 mL of aqueous HCl (6.0 M) and refluxed for 24 h and then cooled to room temperature. The reaction mixture was concentrated in vacuo. To a solution of compound in THF (3.0 mL), saturated NaHCO_3_ (3.0 mL) was added Boc_2_O (151 µL, 0.660 mmol) at 0 °C. The mixture was stirred at room temperature for 24 h. The reaction was diluted with EtOAc, washed with HCl (0.10 M) and brine, dried over Na_2_SO_4_. The residue was purified using silica gel column chromatography (Hexane:EtOAc = 50:50) to afford **7** (45.7 mg, 47%) as a colorless oil:

### 3.4. Pht-Dpv(OTBS)-NH(8-quinoline) ***6***

Amide **4** (840 mg, 2.24 mmol), AgOAc (750 mg, 4.48 mmol), Pd(OAc)_2_ (0.100 g, 0.450 mmol), and *p*-iodophenyl *tert*-butyldimethylsilyl ether (3.00 g, 8.97 mmol) were added to a flask. The mixture was stirred at 90 °C for 6 h under Ar atmosphere. After being cooled to room temperature, the reaction was diluted with AcOEt and then filtered through a pad of celite wash with AcOEt and concentrated in vacuo. The residue was purified by silica gel column chromatography (Hexane:EtOAc = 90:10 then 80:20) to afford **6** (590 mg, 1.02 mmol, 86%) as a white solid: ${\left[\alpha \right]}_{D}^{25}$ −56.7 (*c* 0.45, CHCl_3_); IR (neat) 2929, 1772, 1718, 1530, 1509, 1382, 1260, 914, 826, 787, 719 cm^−1^; ^1^H NMR (400 MHz, CDCl_3_) δ 0.17 (6H, s), 0.86 (3H, d, *J* = 7.3 Hz), 0.97 (9H, s), 2.38 (1H, dd, *J* = 13.7, 10.3 Hz), 3.17 (1H, dd, *J* = 13.7, 3.4 Hz), 3.33–3.35 (1H, m), 4.88 (1H, d, *J* = 10.7 Hz), 6.74 (2H, d, *J* = 7.6 Hz), 7.16 (2H, d, *J* = 7.6 Hz), 7.43 (1H, dd, *J* = 8.3, 4.4 Hz), 7.51 (2H, d, *J* = 3.4 Hz), 7.71 (2H, dd, *J* = 5.4, 2.9 Hz), 7.87 (2H, dd, *J* = 5.4, 2.9 Hz), 8.13 (1H, dd, *J* = 8.3, 1.5 Hz), 8.78 (1H, dd, *J* = 5.4, 3.4 Hz), 8.84 (1H, dd, *J* = 4.4, 1.5 Hz), 10.68 (1H, brs); ^13^C NMR (100 MHz, CDCl_3_) δ 4.5, 15.6, 18.1, 25.6, 34.4, 61.4, 117.0, 119.7, 121.6, 122.0, 123.4, 127.2, 127.8, 130.2, 131.5, 132.1, 134.1, 136.1, 138.7, 148.5, 153.9, 166.5, 168.0; HRMS (ESI) *m*/*z*: [M + Na]^+^; Calcd for C_34_H_37_N_3_O_4_SiNa 602.2447; Found 602.2446.

### 3.5. Boc-Dpv(OH)-OH ***8***

Amide **6** (290 mg, 0.500 mmol) was dissolved in 14 mL of aqueous HCl (6.0 M) was heated at 130 °C in a sealed tube for 24 h and then cooled to room temperature. The reaction mixture was concentrated in vacuo. To a solution of compound in THF (2.5 mL), saturated NaHCO_3_ (2.5 mL) was added Boc_2_O (0.200 mL, 1.00 mmol) at 0 °C. The mixture was stirred at room temperature for 24 h. The solution was diluted with EtOAc, washed with HCl (0.1 M) and brine, dried over Na_2_SO_4_. The residue was purified using silica gel column chromatography (Hexane:EtOAc = 50:50) to afford **8** (120 mg, 0.387 mmol, 77%) as a colorless oil: ${\left[\alpha \right]}_{D}^{25}$ +3.5 (*c* 0.49, CH_3_OH); IR (neat) 3748, 2976, 1702, 1514, 1398, 1244, 1160, 1073, 774, 641 cm^−1^; ^1^H NMR (400 MHz, CDCl_3_) δ 0.86 (3H, brs), 1.47 (9H, s), 2.33–2.43 (1H, m), 2.62–2.66 (1H, m), 4.34 (1H, brs), 5.10 (1H, d, *J* = 9.3 Hz), 6.73 (2H, d, *J* = 7.8 Hz), 6.98 (2H, d, *J* = 7.8 Hz); ^13^C NMR (100 MHz, CD_3_OD) δ 4.7, 28.4, 38.6, 39.8, 57.7, 80.3, 115.9, 129.6, 130.8, 131.8, 156.5, 171.0; HRMS (ESI) *m*/*z*: [M + Na]^+^; Calcd for C_16_H_23_NO_5_Na 332.1471; Found 332.1468.

### 3.6. N_2_-Dml-OH ***13***

Lithium perchlorate (2.12 g, 20.0 mmol), was dissolved in 10 mL anhydrous Et_2_O. TMS-quinine **11** (400 mg, 1.00 mmol) and CH_2_Cl_2_ (20 mL) were added to this solution which was then cooled to −40 °C. DIEA (4.36 mL, 25.0 mmol) and isobutyraldehyde (0.920 mL, 10.0 mmol) were then added to the solution. Propionyl chloride (1.74 mL, 20.0 mmol) was dissolved in CH_2_Cl_2_ (5.0 mL). The solution of propionyl chloride was then added dropwise to the reaction over the course of 3 h. Upon completion of the addition, the reaction was allowed to stir at −40 °C for 16 h. After this time, Et_2_O was added to the solution. The resulting mixture was filtered through a pad of celite and washed with Et_2_O. The solution was concentrated at a light vacuum and diluted with CH_2_Cl_2_. The solution was washed with sat. NH_4_Cl and brine, dried over Na_2_SO_4_ and concentrated at a light vacuum to give crude lactone, which was used in the next step without further purification.

To a solution of the crude lactone in DMSO (30 mL) were added NaN_3_ (1.30 g, 20.0 mmol) and NH_4_Cl (535 mg, 10.0 mmol) at room temperature. The mixture was heated at 50 °C, diluted with aqueous HCl, extracted with EtOAc, washed with brine, dried over Na_2_SO_4_, and concentrated in vacuo. The crude product was purified using silica gel column chromatography (Hexane:EtOAc = 20:80) to afford **14** (718 mg, 4.20 mmol, 42%) as a colorless oil: ${\left[\alpha \right]}_{D}^{20}$ +8.1 (*c* 0.92, CHCl_3_); IR (neat) 2967, 2878, 2157, 2106, 1713, 1419, 893, 852, 663 cm^−1^; ^1^H NMR (400 MHz, CDCl_3_) δ 0.89 (3H, d, *J* = 6.8 Hz), 1.08 (3H, d, *J* = 6.8 Hz), 1.23 (3H, d, *J* = 7.3 Hz), 1.95–2.05 (1H, m), 2.61–2.68 (1H, m), 3.42 (1H, dd, *J* = 8.8, 4.4 Hz); ^13^C NMR (100 MHz, CDCl_3_) δ 14.5, 15.7, 20.5, 29.5, 42.4, 70.6, 180.4; HRMS (ESI) *m*/*z*: [M + Na]^+^; Calcd for C_7_H_13_N_3_O_2_Na 194.0906; Found 194.0910.

### 3.7. N_2_-Dml-OBn ***14***

To a solution of **13** (20.0 mg, 0.120 mmol) in DMF (0.60 mL) were added BnBr (0.0200 mL, 0.180 mmol), NaH (4.00 mg, 0.160 mmol) at 0 °C under Atmosphere. The mixture was stirred at room temperature for overnight, quenched with saturated NaHCO_3_, extracted with EtOAc, washed with brine, dried over Na_2_SO_4_, and concentrated in vacuo. The crude product was purified using silica gel column chromatography (Hexane:EtOAc = 20:80) to afford **14** (20.0 mg, 0.0800 mmol, 64%) as a colorless oil: ${\left[\alpha \right]}_{D}^{18}$ +19.8 (*c* 0.68, CHCl_3_); IR (neat) 2966, 2103, 1734, 1456, 1366, 1341, 1261, 1171, 1147, 1027, 978, 907, 751, 697 cm^−1^; ^1^H NMR (400 MHz, CDCl_3_) δ 0.86 (3H, d, *J* = 6.8 Hz), 1.05 (3H, d, *J* = 6.8 Hz), 1.17 (3H, d, *J* = 7.3 Hz), 1.93–1.99 (1H, m), 2.62–2.69 (1H, m), 3.45 (1H, dd, *J* = 9.3, 3.9 Hz), 5.17 (1H, s), 7.25–7.37 (5H, m); ^13^C NMR (100 MHz, CDCl_3_) δ14.5, 15.5, 20.6, 29.3, 42.7, 66.6, 70.8, 128.27, 128.28, 128.5, 135.7, 174.4; HRMS (ESI) *m*/*z*: [M + Na]^+^; Calcd for C_14_H_19_N_3_O_2_Na 284.1375; Found 284.1377.

### 3.8. Boc-Dml-OBn ***15***

To a solution of **14** (1.52 g, 5.82 mmol) in THF/water (10:1 *v*/*v*, 29 mL) were added Ph_3_P (4.60 g, 17.4 mmol) at 60 °C for 2 h. The reaction solution was cooled to room temperature, concentrated under reduced pressure, and the residue obtained was dissolved in THF/NaHCO_3_ (1:1 *v*/*v*, 17 mL). Cool the solution to 0 °C, add Boc_2_O (1.60 mL, 6.80 mmol), return to ambient temperature and stir overnight. Extracted with EtOAc, washed with brine, dried over Na_2_SO_4_, and concentrated in vacuo. The crude product was purified using silica gel column chromatography (Hexane:EtOAc = 20:80) to afford **15** (1.09 g, 3.40 mmol, 59%) as a colorless oil: ${\left[\alpha \right]}_{D}^{25}$ +18.1 (*c* 0.23, CHCl_3_); IR (neat) 3750, 2974, 2876, 1716, 1507, 1166, 772, 669 cm^−1^; ^1^H NMR (400 MHz, CDCl_3_) δ 0.89 (3H, d, *J* = 6.8 Hz), 0.91 (3H, d, *J* = 6.8 Hz), 1.22 (3H, d, *J* = 6.8 Hz), 1.43 (9H, s), 1.61 (1H, sep, *J* = 6.8 Hz), 2.80-2.87 (1H, m), 3.38 (1H, ddd, *J* = 10.1, 7.3, 4.4 Hz), 5.10 (1H, d, *J* = 15.6 Hz), 5.11 (1H, d, *J* = 15.6 Hz), 5.24 (1H, d, *J* = 10.2 Hz), 7.32 -7.39 (5H, m); ^13^C NMR (100 MHz, CDCl_3_) δ 15.7, 19.2, 19.9, 28.4, 31.8, 40.5, 58.6, 66.3, 78.8, 128.1, 128.3, 128.6, 135.7, 156.4, 175.6; HRMS (ESI) *m*/*z*: [M + Na]^+^ Calcd for C_19_H_29_NO_4_Na 358.1989; Found 358.1992.

### 3.9. Boc-Dpv(OH)-Pro-OBn ***16***

To a solution of **8** (850 mg, 2.70 mmol) and L-proline benzylester (670 mg, 3.24 mmol) in CH_3_CN (13.5 mL) was added EDCI (620 mg, 3.24 mmol), HOAt (440 mg, 3.24 mmol) and NaHCO_3_ (230 mg, 2.70 mmol) under Ar atmosphere. After 24 h of stirring at room temperature, the mixture was concentrated in vacuo. The residue was purified using silica gel column chromatography (Hexane:EtOAc = 50:50) to afford the dipeptide **16** (1.02 g, 2.05 mmol, 76%) as colorless oil: ${\left[\alpha \right]}_{D}^{25}$ −35.4 (*c* 1.20, CHCl_3_); IR (neat) 3326, 2927, 2931, 1744, 1715, 1637, 1514, 1260, 1168, 1016, 753 cm^−1^; ^1^H NMR (400 MHz, CDCl_3,_ mixture of rotamers) δ 0.85 (3H, d, *J* = 6.8 Hz), 1.45 (9H, s), 1.82–1.89 (2H, m), 2.01–2.02 (1H, m), 2.13–2.17 (1H, m), 2.35 (1H, dd, *J* = 13.7, 7.3 Hz), 2.67 (1H, dd, *J* = 13.7, 6.8 Hz), 3.18–3.24 (1H, m), 3.30–3.32 (1H, m), 4.41 (1H, d, *J* = 6.3 Hz), 4.55 (1H, dd, *J* = 8.3, 4.4 Hz), 5.13 (2H, s), 5.30 (1H, d, *J* = 9.8 Hz), 5.91 (1H, brs), 6.74 (2H, d, *J* = 8.3 Hz), 7.05 (2H, d, *J* = 8.3 Hz), 7.28–7.33 (5H, m); ^13^C NMR (100 MHz, CDCl_3,_ mixture of rotamers) δ 14.2, 15.3, 24.9, 28.4, 28.9, 36.3, 38.4, 39.1, 46.5, 58.8, 65.9, 66.9, 115.1, 128.1, 128.3, 128.5, 130.5, 132.2, 135.6, 154.2, 171.9; HRMS (ESI) *m*/*z*: [M + Na]^+^; Calcd for C_28_H_36_N_2_O_6_Na 519.2465; Found 519.2466.

### 3.10. Boc-Dpv(OAc)-Pro-OBn ***17***

To a solution of **16** (520 mg, 1.05 mmol) in CH_2_Cl_2_ (5.25 mL) was added Ac_2_O (120 μL, 1.26 mmol) and DMAP (50.0 mg, 0.42 mmol) under Ar atmosphere. After 2 h of stirring at room temperature, the mixture was concentrated in vacuo. The residue was purified using silica gel column chromatography (Hexane:EtOAc = 50:50) to afford the dipeptide **17** (510 mg, 0.97 mmol, 92%) as colorless oil: ${\left[\alpha \right]}_{D}^{25}$ −38.7 (*c* 1.20, CHCl_3_); IR (neat) 3735, 3308, 2970, 2963, 2931, 1744, 1715, 1651, 1510, 1433, 1168, 753, 711 cm^−1^; ^1^H NMR (400 MHz, CDCl_3,_ mixture of rotamers) δ 0.84 (3H, d, *J* = 6.8 Hz), 1.34 (9H, s), 1.72–1.89 (3H, m), 1.97–2.07 (1H, m), 2.13–2.17 (1H, m), 2.25 (3H, s), 2.35 (1H, dd, *J* = 13.7, 7.3 Hz), 2.67 (1H, dd, *J* = 13.7, 6.8 Hz), 3.17–3.24 (1H, m), 3.29–3.32 (1H, m), 4.41 (1H, d, *J* = 6.3 Hz), 4.55 (1H, dd, *J* = 8.3, 4.4 Hz), 5.13 (2H, s), 5.30 (1H, d, *J* = 9.8 Hz), 5.91 (1H, brs), 6.74 (2H, d, *J* = 8.3 Hz), 7.05 (2H, d, *J* = 8.3 Hz), 7.28–7.33 (5H, m); ^13^C NMR (100 MHz, CDCl_3,_ mixture of rotamers) δ 14.3, 15.3, 24.0, 28.2, 28.9, 36.3, 37.8, 39.1, 46.5, 58.8, 65.9, 67.0, 115.1, 128.1, 128.2, 128.5, 130.5, 132.2, 135.6, 154.2, 172.3; HRMS (ESI) *m*/*z*: [M + Na]^+^; Calcd for C_30_H_39_N_2_O_7_Na 562.2463; Found 562.2467.

### 3.11. Boc-Dpv(OAc)-Pro-Dml-OBn ***20***

To **15** (1.09 g, 3.40 mmol) was added TFA/CH_2_Cl_2_ (1:4 *v*/*v*, 25 mL). After 1 h of stirring at room temperature, the solution was concentrated in vacuo to afford crude 19, which was used in the next step without further purification.

To a solution of **17** (550 mg, 1.05 mmol) in CH_3_OH (5.25 mL) was carefully added Pd(OH)_2_/C (110 mg, 20 wt%) under Ar atmosphere. The solution was purged with H_2_ gas and stirring was continued under H_2_ atmosphere at room temperature for 16 h. The solution was filtered through celite and concentrated in vacuo to afford crude 18, which was used in the next step without further purification.

To a solution of the crude **19** (610 mg, 2.60 mmol) and crude **18** (1.25 g, 2.60 mmol) in CH_3_CN (26 mL) were added DMTMM (740 mg, 2.60 mmol) and Et_3_N (2.17 mL, 15.6 mmol) under Ar atmosphere. After 24 h of stirring at room temperature, the mixture was concentrated in vacuo. The residue was purified using silica gel column chromatography (Hexane:EtOAc = 20:80) to afford **20** (1.01 g, 1.51 mmol, 58%) as a colorless oil: ${\left[\alpha \right]}_{D}^{25}$ +8.8 (*c* 2.40, CHCl_3_); IR (neat) 3734, 3413, 3309, 2973, 2877, 1715, 1507, 1366, 1168, 754 cm^−1^; ^1^H NMR (400 MHz, CDCl_3,_ mixture of rotamers) δ 0.85 (3H, d, *J* = 6.8 Hz), 0.86 (3H, d, *J* = 6.8 Hz), 0.88 (3H, d, *J* = 6.8 Hz), 1.17 (3H, d, *J* = 7.3 Hz), 1.46 (9H, s), 1.89 (2H, t, *J* = 5.4 Hz), 1.98–2.09 (2H, m), 2.17–2.23 (1H, m), 2.26 (3H, s), 2.48 (1H, dd, *J* = 13.7, 6.8 Hz), 2.74 (1H, dd, *J* = 13.2, 7.8 Hz), 2.86 (1H, dd, *J* = 6.8, 3.4 Hz), 3.15–3.18 (1H, m), 3.23–3.28 (1H, m), 3.68 (1H, td, *J* = 9.8, 3.4 Hz), 4.47–4.52 (2H, m), 5.01 (1H, d, *J* = 12.2 Hz), 5.06 (2H, d, *J* = 12.2 Hz), 5.37 (1H, d, *J* = 9.8 Hz), 6.80 (1H, d, *J* = 10.7 Hz), 6.98 (2H, d, *J* = 8.8 Hz), 7.29–7.37 (7H, m); ^13^C NMR (100 MHz, CDCl_3,_ mixture of rotamers) δ 0.3, 14.5, 16.2, 19.8, 20.1, 21.4, 25.2, 28.6, 28.7, 29.4, 32.2, 38.9, 40.0, 47.0, 53.8, 57.3, 61.0, 66.6, 79.8, 121.7, 128.3, 128.6, 128.9, 130.8, 135.9, 138.4, 149.3, 169.7, 171.8, 172.1, 176.3; HRMS (ESI) *m*/*z*: [M + Na]^+^; Calcd for C_37_H_51_N_3_O_8_Na 688.3566; Found 688.3568.

### 3.12. Boc-Dpv(OH)-Pro-Dml-OBn ***21***

To a solution of **20** (184 mg, 0.276 mmol) in CH_3_OH (0.14 mL) were added TEA (7.00 µL, 0.502 µmol) under Ar atmosphere. After 24 h of stirring at room temperature, the mixture was concentrated in vacuo. The crude product was purified using silica gel column chromatography (Hexane:Acetone = 75:25) to afford **21** (155 mg, 0.0205 mmol, 90%) as a colorless foam: ${\left[\alpha \right]}_{D}^{23}$ +6.3 (*c* 1.18, CHCl_3_); IR (neat) 3403, 3311, 3009, 2975, 2935, 2878, 1715, 1652, 1615, 1594, 1516, 1455, 1391, 1367, 1235, 1172, 1101, 1057, 1003, 877, 826, 755, 698, 666 cm^−1^; ^1^H NMR (400 MHz, CDCl_3_, mixture of rotamers) δ 0.72–0.94 (9H, m), 1.20 (3H, d, *J* = 7.3 Hz), 1.35–1.58 (10H, m), 1.70–1.92 (2H, m), 1.96–2.10 (4H, m), 2.15–2.25 (1H, m), 2.40 (1H, dd, *J* = 13.7, 6.8 Hz), 2.59–2.69 (1H, m), 2.81–2.92 (1H, m), 3.21–3.35 (2H, m), 3.69 (1H, td, *J* = 9.76, 3.4 Hz), 4.45–4.55 (2H, m), 5.06 (2H, s), 5.38 (1H, d, *J* = 9.8 Hz), 6.74 (2H, d, *J* = 8.3 Hz), 6.90 (1H, d, *J* = 10.2 Hz), 7.06 (2H, d, *J* = 7.8 Hz), 7.18–7.41 (5H, m); ^13^C NMR (100 MHz, CDCl_3_, mixture of rotamers) δ 13.9, 15.8, 19.6, 19.8, 24.9, 28.3, 29.1, 31.8, 38.0, 39.5, 39.7, 46.7, 53.7, 57.2, 60.6, 66.3, 79.8, 115.1, 128.0, 128.1, 128.3, 128.5, 128.6, 130.1, 130.3, 131.1, 135.5, 155.0, 156.2, 171.9, 172.1, 175.9; HRMS (ESI) *m*/*z*: [M + Na]^+^ Calcd for C_35_H_49_N_3_O_7_Na 646.3478; Found 646.3463

### 3.13. Boc-Dpv(OBn)-Pro-Dml-OBn ***22***

To a solution of **21** (12.1 mg, 0.0194 mmol) in CH_3_CN (0.16 mL) were added K_2_CO_3_ (8.90 mg, mmol), BnBr (8.00 µL, 0.0669 mmol) and KI (1.10 mg, 6.63 µmol) under Ar atmosphere at room temperature. The mixture was stirred for 24 h, quenched with sat. NaHCO_3_, extracted with EtOAc, washed with brine, dried over Na_2_SO_4_, filtered, and concentrated in vacuo. The crude product was purified using silica gel column chromatography (Hexane:Acetone = 80:20) to afford **22** (14.6 mg, 0.0205 mmol, 95%) as a colorless oil: ${\left[\alpha \right]}_{D}^{29}$ +13.1 (*c* 1.22, CHCl_3_); IR (neat) 3416, 3315, 2972, 2932, 2876, 1714, 1652, 1507, 1456, 1417, 1392, 1367, 1241, 1172, 1099, 1026, 907, 808, 751, 697, 666 cm^−1^; ^1^H NMR (400 MHz, CDCl_3_, mixture of rotamers) δ 0.72–0.95 (9H, m), 1.17 (3H, d, *J* = 7.3 Hz), 1.35–1.52 (10H, m), 1.79–2.23 (5H, m), 2.44 (1H, dd, *J* = 13.7, 6.8 Hz), 2.61–2.75 (1H, m), 2.80–2.92 (1H, m), 3.20–3.39 (2H, m), 3.69 (1H, td, *J* = 10.1, 3.4 Hz), 4.41–4.59 (2H, m), 4.92–5.08 (4H, m), 5.35 (1H, d, *J* = 8.8 Hz), 6.72–6.95 (3H, m), 7.15–7.50 (12H, m); ^13^C NMR (100 MHz, CDCl_3_, mixture of rotamers) δ 14.0, 15.9, 19.5, 19.8, 24.9, 29.2, 29.6, 31.7, 31.8, 38.4, 39.6, 39.7, 46.7, 53.4, 53.8, 53.8, 57.0, 60.6, 66.3, 69.9, 79.5, 114.6, 127.3, 127.8, 128.0, 128.3, 128.5, 128.6, 130.4, 132.7, 135.6, 137.1, 155.9, 157.1, 171.5, 172.0, 175.9; HRMS (ESI) *m*/*z*: [M + Na]^+^ Calcd for C_42_H_55_N_3_O_7_Na 736.3932; Found 736.3931.

### 3.14. BnO-Lac-Pro-O-Hiv-D-MeVal-Pro-Dpv(OAc)-Pro-Dml-OBn ***23***

To **20** (380 mg, 0.570 mmol) was added TFA/CH_2_Cl_2_ (1:4 *v/v*, 19 mL). After 1 h of stirring at room temperature, the solution was concentrated in vacuo to afford crude amine, which was used in the next step without further purification.

To a solution of the crude amine and **2** (340 mg, 0.570 mmol) in CH_3_CN (5.70 mL) were added Et_3_N (0.480 mL, 3.42 mmol) and DMTMM (160 mg, 0.570 mmol) under Ar atmosphere. After 24 h of stirring at room temperature, the solution was concentrated in vacuo. The residue was purified using silica gel column chromatography to afford **23** (368 mg, 0.325 mmol, 57% over 2 steps) as a colorless foam: ${\left[\alpha \right]}_{D}^{25}$ −12.7 (*c* 1.20, CHCl_3_); IR (neat) 3733, 3410, 3309, 2973, 2965, 2876, 1730, 1663, 1510, 1430, 1183, 1100, 753 cm^−1^; ^1^H NMR (400 MHz, CDCl_3_, mixture of rotamers) δ 0.69–1.17 (20H, m), 1.24–1.27 (4H, m), 1.38–1.59 (4H, m), 1.70–2.16 (9H, m), 2.28 (3H, s), 2.23–2.94 (11H, m), 3.08 (3H, s), 3.10–3.77 (5H, m), 4.05–4.93 (11H, m), 6.78 (0.5H, d, *J* = 10.3 Hz), 6.95 (1.5H, d, *J* = 8.3 Hz), 7.23–7.35 (14H, m); ^13^C NMR (100 MHz, CDCl_3_, mixture of rotamers) δ13.8, 14.2, 15.83, 15.85, 16.0, 16.6, 17.0, 17.2, 17.8, 18.0, 19.2, 19.4, 19.60, 19.67, 19.7, 20.0, 20.9, 21.0, 24.6, 24.9, 25.5, 26.3, 29.0, 29.8, 30.0, 31.7, 31.8, 38.6, 39.5, 46.6, 46.7, 56.8, 58.6, 59.1, 59.9, 60.5, 60.6, 66.15, 66.19, 70.8, 71.0, 74.8, 75.6, 121.1, 121.2, 127.56, 127.59, 127.77, 127.82, 127.84, 128.23, 128.25, 128.27, 128.4, 128.5, 130.3, 130.4, 135.4, 137.6, 137.9, 148.8, 167.5, 169.2, 169.7, 170.9, 171.0, 171.2, 171.3, 171.5, 171.6, 175.86, 175.88; HRMS (ESI) *m*/*z*: [M + Na]^+^; Calcd for C_63_H_86_N_13_O_6_Na 1157.3893; Found 1157.6145.

### 3.15. Dolastatin 16 Acetate ***24***

To a solution of **23** (110 mg, 0.100 mmol) in CH_3_OH (1.00 mL) was carefully added Pd(OH)_2_/C (22.0 mg, 20 wt%) under Ar atmosphere. The solution was purged with H_2_ gas and stirring was continued under H_2_ atmosphere at room temperature for 16 h. The solution was filtered through celite and concentrated in vacuo to afford crude carboxylic acid, which was used in the next step without further purification.

A solution of the crude carboxylic acid **10a** (9.50 mg, 0.0100 mmol) in CH_3_CN (6.3 mL) was dropwised to a refluxing solution of 2-chloro-1-methylpyridinium iodide (13.0 mg, 0.0130 mmol) and Et_3_N (0.0150 mL, 0.110 mmol) in CH_3_CN (3.1 mL) over a 3 h period via addition funnel. The addition funnel was rinsed with a total of 0.6 mL of CH_3_CN. The mixture was refluxed for overnight. After being cooled to ambient temperature, the mixture was concentrated in vacuo. The residue was purified by silica gel column chromatography on silica gel (Hexane:EtOAc = 20:80) to afford **24** as a colorless oil (4.90 mg, 0.005 mmol, 64% over 2 steps): ${\left[\alpha \right]}_{D}^{25}$ +29.4 (*c* 0.38, CHCl_3_); IR (neat) 3394, 3324, 2965, 2876, 1750, 1732, 1650, 1507, 1458, 1426, 1386, 1298, 1194, 1090, 1019, 753, 666 cm^−1^; ^1^H NMR (400 MHz, CDCl_3_) δ 0.82–0.93 (14H, m), 1.01–1.09 (9H, m), 1.43 (3H, d, *J* = 6.8 Hz), 1.45–1.60 (2H, m), 1.65–2.26 (9H, m), 2.28 (3H, s), 2.30–2.44 (6H, m), 2.45–2.55 (2H, m), 2.78–2.90 (2H, m), 3.08 (3H, s), 3.35–3.50 (2H, m), 3.60–3.70 (2H, m), 3.85–3.92 (1H, m), 4.44 (1H, d, *J* = 6.8 Hz), 4.54 (1H, d, *J* = 7.8 Hz), 4.60–4.64 (1H, m), 4.94 (1H, d, *J* = 8.8 Hz), 5.12–5.20 (2H, m), 5.41 (1H,d, *J* = 2.9 Hz), 6.72 (1H, d, *J* = 8.8 Hz), 6.74 (1H, d, *J* = 8.7 Hz), 7.00 (2H, d, *J* = 8.3 Hz), 7.41 (2H, d, *J* = 8.8 Hz), 7.68 (1H, d, *J* = 10.2 Hz); ^13^C NMR (100 MHz, CDCl_3_) δ 14.8, 15.2, 16.0, 17.1, 17.7, 19.6, 19.7, 20.3, 21.1, 21.7, 24.7, 24.8, 24.9, 25.4, 25.5, 28.2, 29.5, 30.6, 30.7, 32.2, 38.6, 41.0, 45.9, 46.4, 47.5, 50.2, 56.2, 57.8, 58.8, 59.4, 59.5, 61.2, 66.6, 120.1, 121.4, 130.6, 138.2, 149.0, 161.8, 169.0, 169.50, 169.54, 170.95, 170.99, 171.1, 172.3, 174.6; HRMS (ESI) *m*/*z*: [M + Na]^+^; Calcd for C_49_H_72_N_6_O_12_Na 959.5108; Found 959.5100.

### 3.16. BnO-Lac-Pro-O-Hiv-D-MeVal-Pro-OBn ***25***

To a solution of **2** (6.3 mg, 0.0107 mmol) in CH_2_Cl_2_ (0.30 mL) were added BnOH (1.30 μL, 0.0126 mmol), DMAP (catalytic) and EDCI (3.00 mg, 0.0156 mmol) under Ar atmosphere. The mixture was stirred for 27 h at room temperature and concentrated in vacuo. The crude product was purified using column chromatography (5% EtOAc in hexane) to afford **25** (4.70 mg, 0.00694 mmol, 64%) as a colorless oil:

${\left[\alpha \right]}_{D}^{21}$ +35.1 (*c* 0.47, CHCl_3_); IR (neat) 2963, 2927, 2874, 1742, 1646, 1454, 1426, 1370, 1295, 1255, 1186, 1092, 1014, 738, 699 cm^−1^; ^1^H NMR (CDCl_3_, 400 MHz, mixture of rotamers) δ 0.68–1.01 (12H, m), 1.33–1.42 (3H, m), 1.70–2.32 (10H, m), 2.82 (2H, s), 2.95 (1H, s), 3.30–3.72 (4H, m), 4.10–4.35 (2H, m), 4.40–4.75 (3H, m), 4.90–5.10 (4H, m), 7.19–7.31 (10H, m); ^13^C NMR (CDCl_3_, 100 MHz, mixture of rotamers) δ 14.1, 15.9, 16.1, 17.1, 17.2, 17.8, 19.7, 19.8, 20.1, 24.9, 25.1, 26.2, 26.3, 28.0, 28.8, 28.90, 28.99, 29.6, 29.81, 29.85, 46.52, 46.56, 58.5, 58.8, 59.1, 60.1, 66.7, 67.1, 70.9, 72.6, 75.2, 75.4, 77.2, 127.6, 127.72, 127.75, 128.1, 128.2, 128.31, 128.38, 128.52, 128.58, 135.5, 137.7, 167.8, 168.1, 169.1, 169.6, 171.3, 171.71, 171.78, 172.0, 172.3; HRMS (ESI) *m*/*z*: [M + Na]^+^ Calcd for C_38_H_51_N_3_O_8_Na 700.3574; Found 700.3578.

### 3.17. Boc-Pro-O-Hiv-D-MeVal-Pro-CH_2_OBn ***29***

To peptide **27^45^** (51.4 mg, 0.127 mmol) was added TFA/DCM (1:4 *v*/*v*, 2.2 mL). After 1 h of stirring at room temperature, the mixture was concentrated in vacuo. The residue TFA salt was added 0.5 M NaOH aq, extracted with DCM, washed with brine, dried over Na_2_SO_4_, filtered, and concentrated in vacuo to afford crude amine, which was used in the next step without further purification.

To a solution of the crude amine in MeCN (0.65 mL) was added 4 N HCl in dioxane (31.0 µL, 0.127 mmol) under Ar atmosphere. After 30 min of stirring at room temperature, to the mixture were added **28** (51.5 mg, 0.127 mmol), PyBroP (71.1 mg, 0.152 mmol) and *i*Pr_2_NEt (66.0 µL, 0.381 mmol) The mixture was stirred for 24 h, quenched with *sat.* NH_4_Cl, extracted with EtOAc, washed with brine, dried over Na_2_SO_4_, filtered, and concentrated in vacuo. The crude product was purified using silica gel column chromatography (Hexane:Acetone = 80:20) to afford **29** (56.1 mg, 0.0932 mmol, 73%) as a colorless oil: ${\left[\alpha \right]}_{D}^{21}$ +18.0 (*c* 1.03, CHCl_3_); IR (neat), 2970, 2933, 2875, 1747, 1701, 1643, 1454, 1397, 1365, 1297, 1254, 1188, 1168, 1119, 1088, 1029, 1011, 917, 890, 772, 740, 699 cm^−1^; ^1^H NMR (400 MHz, CDCl_3_, mixture of rotamers) δ 0.80 (3H, t, *J* = 7.3 Hz), 0.85–1.13 (9H, m), 1.32–1.52 (9H, m), 1.69–2.41 (10H, m), 2.89 (1.9H, s), 2.97 (0.5H, s), 2.99 (0.6H, s), 3.25–3.59 (5.5H, m), 3.60–3.70 (0.5H, m), 4.23–4.38 (1.5H, m), 4.40–4.60 (2.5H, m), 4.94 (0.6H, d, *J* = 10.7 Hz), 4.95–5.05 (1H, m), 5.20 (0.4H, dd, *J* = 10.7, 8.3), 7.22–7.40 (5H, m); ^13^C NMR (100 MHz, CDCl_3_, mixture of rotamers) δ 16.3, 16.3, 16.4, 18.1, 18.2, 19.5, 19.6, 19.6, 19.7, 19.8, 20.0, 20.0, 21.7, 23.2, 24.0, 24.0, 26.5, 26.5, 27.4, 28.1, 28.3, 28.5, 28.5, 28.8, 29.0, 29.1, 29.6, 29.9, 29.9, 29.9, 30.4, 30.5, 45.7, 46.3, 46.3, 46.9, 55.5, 56.6, 56.6, 58.2, 58.5, 58.5, 59.5, 60.3, 60.3, 69.6, 71.3, 72.8, 73.0, 75.1, 75.2, 75.5, 79.6, 79.6, 79.7, 127.4, 127.4, 127.5, 127.6, 128.3, 128.4, 138.6, 153.9, 167.8, 168.0, 169.2, 127.8; HRMS (ESI) m/z: [M + Na]^+^ Calcd for C_33_H_51_O_7_N_3_Na 524.3614; Found 524.3619.

### 3.18. Antifouling Assay

Antifouling assay against larvae of the barnacle *Amphibalanus amphitrite* was conducted according to the previous literature [18,19,38]. The adult barnacles, *A.* *amphitrite*, obtained from oyster farms in Lake Hamana and a pier of Shimizu bay, Shizuoka, were kept in an aquarium at 20 °C and were fed on *Artemia salina* nauplii. Broods were released as I–II stage nauplii upon immersion in seawater after drying overnight. The nauplii (1.0~3.0 indiv./mL) thus obtained were cultured in 2.0 L filtered (0.2 μm) natural seawater (diluted by DW: salinity 28) containing penicillin G (20 μg/mL) and streptomycin sulfate (30 μg/mL) at 25 °C and were fed on the diatom *Chaetoceros gracillis* at concentrations of 40 × 10^4^ cells/mL. Larvae reached the cyprid stage in 5 days. The cyprids were collected, then stored at 4 °C until use (0-day-old).

The test compounds were dissolved in ethanol and aliquots of the solution (20 μL) were transferred to wells of a 24-well polystyrene culture plates and then air-dried for 3 h at room temperature and CuSO_4_ was used as positive compound. Four wells were used for each concentration (0.03, 0.1, 0.3, 1.0, 3.0, 10.0 μg/mL). To each well were added filtered (0.2 mm) natural seawater (2.0 mL, salinity 28) and six 2-day-old cyprids. The plates were kept in the dark at 25 °C for 48 h. The numbers of cyprids that attached, metamorphosed, died, or did not settle were counted under a microscope. Three or four trials were carried out for each concentration. Antifouling activity (EC_50_) indicates the concentration reducing the larval settlement to 50% of the control (non-treatment) by Probit analysis. Toxicity of compounds were expressed as LC_50_ value, which indicates the concentration showing 50% mortality estimated by Probit analysis. If mortality rate did not show over 50% at most hagh concentration (10.0 μg/mL), then LC_50_ value was indicated as over 10.0 μg/mL.

## 4. Conclusions

In summary, we have developed new methodologies for the derivatives of the two unusual amino acids found in dolastatin 16 through a C-H activation reaction for dolaphenvaline and an enantio- and diastereoselective [2+2] addition reaction for dolamethylleuine. These synthetic routes enabled effective access to especially the southern fragment of dolastatin 16. Many trends of the derivatives towards antifouling activity were exhibited. Specifically, less polar small fragments showed strong antifouling activity against the cypris larvae of *A. amphitrite* without detectable toxicity, although the whole structure was required for extremely potent activity. These results will be useful toward the development of green antifouling materials, and further studies are in progress in our laboratory.

## Figures and Tables

**Table 1:** Antifouling activities of synthetic samples against cypris larvae of *A. amphitrite*.

Compound	EC_50_ (μg/mL) ^1^	EC_50_ (μM)	LC_50_ (μg/mL) ^2^
**1** **^3^**	<0.03	<0.03	>10
**2** **^3^**	>10	>17.0	>10
**3** **^3^**	1.17	1.92	>10
**24**	1.74	1.86	>10
**Boc-3**	0.79	1.30	>10
**21**	0.60	0.96	>10
**22**	4.62	6.47	>10
**25**	0.90	1.32	>10
**29**	3.27	6.52	>10
**CuSO_4_ ^3^**	0.10	0.63	>10

^1^ EC_50_ (50% effective concentration), ^2^ LC_50_ (50% leathal concentration),**^3^** according to [38].

## Data Availability

Data are contained within the article or Supplementary Materials.

## Supplementary Materials

The following are available online at www.mdpi.com/article/10.3390/md20020124/s1, NMR spectra of synthetic samples. Figure S1: ^1^H and ^13^C NMR spectra of compound **5**. Figure S2: ^1^H and ^13^C NMR spectra of compound **6**. Figure S3: ^1^H and ^13^C NMR spectra of compound **8**. Figure S4: ^1^H and ^13^C NMR spectra of compound **13**. Figure S5: ^1^H and ^13^C NMR spectra of compound **14**. Figure S6: ^1^H and ^13^C NMR spectra of compound **16**. Figure S7: ^1^H and ^13^C NMR spectra of compound **17**. Figure S8: ^1^H and ^13^C NMR spectra of compound **20**. Figure S9: ^1^H and ^13^C NMR spectra of compound **21**. Figure S10: ^1^H and ^13^C NMR spectra of compound **22**. Figure S11: ^1^H and ^13^C NMR spectra of compound **23**. Figure S12: ^1^H and ^13^C NMR spectra of compound **24**. Figure S13: ^1^H and ^13^C NMR spectra of compound **25**. Figure S14: ^1^H and ^13^C NMR spectra of compound **29**.

## Abbreviations

EDCI	1-ethyl-3-(3-dimethylaminopropyl)carbodiimide hydrochloride,
HOAt	1-hydroxy-7-azabenzotriazole,
DMTMM	4-(4,6-dimethoxy-1,3,5-triazin-2-yl)-4-methylmorpholinium tetrafluoroborate,
PyBrop	bromotripyrrolidinophosphonium hexafluorophosphate.

## References

[1] Pettit G.R., Xu J.-P., Hogan F., Williams M.D., Doubek D.L., Schmidt J.M., Cerny R.L., Boyd M.R. (1997). Isolation and Structure of the Human Cancer Cell Growth Inhibitory Cyclodepsipeptide Dolastatin 16. J. Nat. Prod..

[2] Pettit G.R., Smith T.H., Xu J.-P., Herald D.L., Flahive E.J., Anderson C.R., Belcher P.E., Knight J.C. (2011). Antineoplastic Agents. 590. X-ray Crystal Structure of Dolastatin 16 and Syntheses of the Dolamethylleuine and Dolaphenvaline Units. J. Nat. Prod..

[3] Pettit G.R., Smith T.H., Arce P.M., Flahive E.J., Anderson C.R., Chapuis J.-C., Xu J.-P., Groy T.L., Belcher P.E., Macdonald C.B. (2015). Antineoplastic Agents. 599. Total Synthesis of Dolastatin 16. J. Nat. Prod..

[4] Tan L.K., Goh B.P.L., Tripathi A., Lim M.G., Dickinson G.H., Lee S.S.C., Teo S.L.M. (2010). Natural antifoulants from the marine cyanobacterium Lyngbya majuscule. Biofouling.

[5] Brooks S.J., Waldock M., Arai T., Harino H., Ohji M., Langston W.J. (2009). Copper Biocides in the Marine Environment. Ecotoxicology of Antifouling Biocides.

[6] Shimasaki Y., Kitano T., Oshima Y., Inoue S., Imada N., Honjo T. (2003). Tributyltin causes masculinization in fish. Environ. Toxicol. Chem..

[7] McAllister B.G., Kime D.E. (2003). Early life exposure to environmental levels of the aromatase inhibitor tributyltin causes masculinisation and irreversible sperm damage in zebrafish (Danio rerio). Aquat. Toxicol..

[8] Weis J.S., Perlmutter J. (1987). Effects of Tributyltin on Activity and Burrowing Behavior of the Fiddler Crab, Uca pugilator. Estuaries.

[9] Weis J.S., Kim K. (1988). Tributyltin is a teratogen in producing deformities in limbs of the fiddler crab,Uca pugilator. Arch. Environ. Contam. Toxicol..

[10] Horiguchi T., Shiraishi H., Shimizu M., Yamazaki S., Morita M. (1995). Imposex in Japanese gastropods (neogastropoda and mesogastropoda): Effects of tributyltin and triphenyltin from antifouling paints. Mar. Pollut. Bull..

[11] Terlizzi A., Delos A.L., Garaventa F., Faimali M., Gerace S. (2004). Limited effectiveness of marine protected areas: Imposex in Hexaplex trunculus (Gastropoda, Muricidae) populations from Italian marine reserves. Mar. Pollut. Bull..

[12] Gibbs P.E., Bryan G.W. (1986). Reproductive Failure in Populations of the Dog-Whelk, Nucella Lapillus, Caused by Imposex Induced by Tributyltin from Antifouling Paints. J. Mar. Biol. Assoc. UK.

[13] Gibbs P.E., Bryan G.W., de Mora S.J. (1996). TBT-Induced Imposex in Neogastropod Snails: Masculinization to Mass Extinction. Tributyltin: Case Study of an Environmental Contaminant.

[14] Horiguchi T., Arai T., Harino H., Ohji M., Langston W.J. (2009). Mechanism of Imposex Induced by Organotins in Gastropods. Ecotoxicology of Antifouling Biocides.

[15] Evans S.M. (1999). TBT or not TBT?: That is the question. Biofouling.

[16] Konstantinou I.K., Albanis T.A. (2004). Worldwide occurrence and effects of antifouling paint booster biocides in the aquatic environment: A review. Environ. Int..

[17] Thomas K.V., Brooks S. (2010). The Environmental Fate and Effects of Antifouling Paint Biocides. Biofouling.

[18] Kitano Y., Ito T., Suzuki T., Nogata Y., Shinshima K., Yoshimura E., Chiba K., Tada M., Sakaguchi I. (2002). Synthesis and Antifouling Activity of 3-Isocyanotheonellin and Its Analogues. J. Chem. Soc. Perkin Trans..

[19] Nogata Y., Kitano Y., Yoshimura E., Shinshima K., Sakaguchi I. (2004). Antifouling Activity of Simple Synthetic Isocyanides Against Larvae of the Barnacle *Balanus amphitrite*. Biofouling.

[20] Kitano Y., Akima C., Yoshimura E., Nogata Y. (2011). Anti-barnacle activity of novel simple alkyl isocyanides derived from citronellol. Biofouling.

[21] Kitano Y., Chiba K., Tada M. (2001). Highly Efficient Conversion of Alcohols to Isocyanides. Synthesis.

[22] Mihara K., Okada I., Chiba K., Kitano Y. (2014). Facile Synthesis of *N*-Substituted Amides from Alkenes and Amides by a Brønsted Acid Mediated Electrophilic Addition Reaction. Synthesis.

[23] Fukuda T., Wagatsuma H., Kominami Y., Nogata Y., Yoshimura E., Chiba K., Kitano Y. (2016). Anti-barnacle Activity of Isocyanides Derived from Amino Acids. Chem. Biodivers..

[24] Inoue Y., Takashima S., Nogata Y., Yoshimura E., Chiba K., Kitano Y. (2018). Isocyanides Derived from *α*,*α*-Disubstituted Amino Acids: Synthesis and Antifouling Activity Assessment. Chem. Biodivers..

[25] Takamura H., Ohashi T., Kikuchi T., Endo N., Fukuda Y., Kadota I. (2017). Late-stage divergent synthesis and antifouling activity of geraniol–butenolide hybrid molecules. Org. Biomol. Chem..

[26] Takamura H., Kikuchi T., Endo N., Fukuda Y., Kadota I. (2016). Total Synthesis of Sarcophytonolide H and Isosarcophytonolide D: Structural Revision of Isosarcophytonolide D and Structure−Antifouling Activity Relationship of Sarcophytonolide H. Org. Lett..

[27] Sjögren M., Johnson A.-L., Hedner E., Dahlström M., Göransson U., Shirani H., Bergman J., Jonsson P.R., Bohlin L. (2006). Antifouling Activity of Synthesized Peptide Analogs of the Sponge Metabolite Barettin. Peptides.

[28] Qian P.-Y., Chen L., Xu Y. (2013). Mini-review: Molecular Mechanisms of Antifouling Compounds. Biofouling.

[29] Liao S., Xu Y., Tang Y., Wang J., Zhou X., Xu L., Liu Y. (2015). Design, Synthesis and Biological Evaluation of Soluble 2,5-Diketopiperazines Derivatives as Potential Antifouling Agents. RSC Adv..

[30] Fusetani N. (2011). Antifouling marine natural products. Nat. Prod. Rep..

[31] Nishikawa K., Nakahara H., Shirokura Y., Nogata Y., Yoshimura E., Umezawa T., Okino T., Matsuda F. (2010). Total Synthesis of 10-Isocyano-4-cadinene and Determination of Its Absolute Configuration. Org. Lett..

[32] Nishikawa K., Nakahara H., Shirokura Y., Nogata Y., Yoshimura E., Umezawa T., Okino T., Matsuda F. (2011). Total Synthesis of 10-Isocyano-4-cadinene and Its Stereoisomers and Evaluation of Antifouling Actitities. J. Org. Chem..

[33] Nishikawa K., Umezawa T., Garson M.J., Matsuda F. (2012). Confirmation of the Configurations of 10-Isothiocyano-4-cadinene through Synthesis. J. Nat. Prod..

[34] Umezawa T., Oguri Y., Matsuura H., Yamazaki S., Suzuki M., Yoshimura E., Furuta T., Nogata Y., Serisawa Y., Matsuyama-Serisawa K. (2014). Omaezallene from Red Alga Laurencia sp.: Structure Elucidation, Total Synthesis and Antifouling Activity. Angew. Chem. Int. Ed..

[35] Umezawa T., Prakoso N.I., Kannaka M., Nogata Y., Yoshimura E., Okino T., Matsuda F. (2019). Synthesis and Structure-Activity Relationship of Omaezallene Derivatives. Chem. Biodivers..

[36] Umezawa T., Mizutani N., Matsuo K., Tokunaga Y., Matsuda F., Nehira T. (2021). Assignment of Absolute Configuration of Bromoallenes by Vacuum-Ultraviolet Circular Dichroism (VUVCD). Molecules.

[37] Umezawa T., Sato A., Ameda Y., Casalme L.O., Matsuda F. (2015). Synthetic Study on Dolastatin 16: Concise and Scalable Synthesis of Two Unusual Amino Acid Units. Tetrahedron Lett..

[38] Casalme L.O., Yamauchi A., Sato A., Petitbois J.G., Nogata Y., Yoshimura E., Okino T., Umezawa T., Matsuda F. (2017). Total Synthesis and Biological Activity of Dolastatin 16. Org. Biomol. Chem..

[39] Reddy B.V.S., Reddy L.R., Corey E.J. (2006). Novel Acetoxylation and C−C Coupling Reactions at Unactivated Positions in α-Amino Acid Derivatives. Org. Lett..

[40] Zhu C., Shen X., Nelson S.G. (2004). Cinchona Alkaloid-Lewis Acid Catalyst Systems for Enantioselective Ketene−Aldehyde Cycloadditions. J. Am. Chem. Soc..

[41] Shiina I., Kubota M., Ibuka R. (2002). A Novel and Efficient Macrolactonization of ω-Hydroxycarboxylic Acids Using 2-Methyl-6-nitrobenzoic Anhydride (MNBA). Tetrahedron Lett..

[42] Shiina I., Kubota M., Oshiumi H., Hashizume M. (2004). An Effective Use of Benzoic Anhydride and Its Derivatives for the Synthesis of Carboxylic Esters and Lactones:  A Powerful and Convenient Mixed Anhydride Method Promoted by Basic Catalysts. J. Org. Chem..

[43] Shiina I., Fukui H., Sasaki A. (2007). Synthesis of Lactones Using Substituted Benzoic Anhydride as a Coupling Reagent. Nat. Protoc..

[44] Narasaka K., Maruyama K., Mukaiyama T. (1978). A Useful Method for the Synthesis of Macrocyclic Lactone. Chem. Lett..

[45] Aurelio L., Brownlee R.T.C., Hughes A.B. (2008). Solution-Phase Peptide Synthesis; Synthesis of ‘North-Western’ and ‘South-Eastern’ Fragments of the Antifungal Cyclodepsipeptide Petriellin A. Aust. J. Chem..

