# Supplementary material for: Effective Synthesis and Antifouling Activity of Dolastatin 16 Derivatives
Source: Mar Drugs. 2022 Feb 4;20(2):124. doi: 10.3390/md20020124 (PMC8876244; doi:10.3390/md20020124)

Effective Synthesis and Antifouling Activity of Dolastatin 16 Derivatives

Loida O. Casalme[1], Keisuke Katayama[1], Yoshiki Hayakawa[1], Kensuke Nakamura[1], Arisa Yamauchi[1], Yasuyuki Nogata[2], Erina Yoshimura[3], Fuyuhiko Matsuda[1], Taiki Umezawa[1]*

[1]Division of Environmental Materials Science, Graduate School of Environmental Science, Hokkaido University, N10W5 Sapporo 060-0810, Japan, e-mail: umezawa@ees.hokudai.ac.jp,

[2] Sustainable System Research Laboratory, Central Research Institute of Electric Power Industry, 1646 Abiko, Abiko, Chiba 270-1194, Japan

[3] CERES, Inc., 1-4-5 Midori, Abiko, Chiba 270-1153, Japan

Table of Contents

Figure S1. ^{1}H and ^{13}C NMR spectra of compound **5** 2

Figure S2. ^{1}H and ^{13}C NMR spectra of compound **6** 3

Figure S3. ^{1}H and ^{13}C NMR spectra of compound **8** 4

Figure S4. ^{1}H and ^{13}C NMR spectra of compound **13** 5

Figure S5. ^{1}H and ^{13}C NMR spectra of compound **14** 6

Figure S6. ^{1}H and ^{13}C NMR spectra of compound **16** 7

Figure S7. ^{1}H and ^{13}C NMR spectra of compound **17** 8

Figure S8. ^{1}H and ^{13}C NMR spectra of compound **20** 9

Figure S9. ^{1}H and ^{13}C NMR spectra of compound **21** 10

Figure S10. ^{1}H and ^{13}C NMR spectra of compound **22** 11

Figure S11. ^{1}H and ^{13}C NMR spectra of compound **23** 12

Figure S12. ^{1}H and ^{13}C NMR spectra of compound **24** 13

Figure S13. ^{1}H and ^{13}C NMR spectra of compound **25** 14

Figure S14. ^{1}H and ^{13}C NMR spectra of compound **29** 15

Figure S1. 1H and 13C NMR spectra of compound **5**

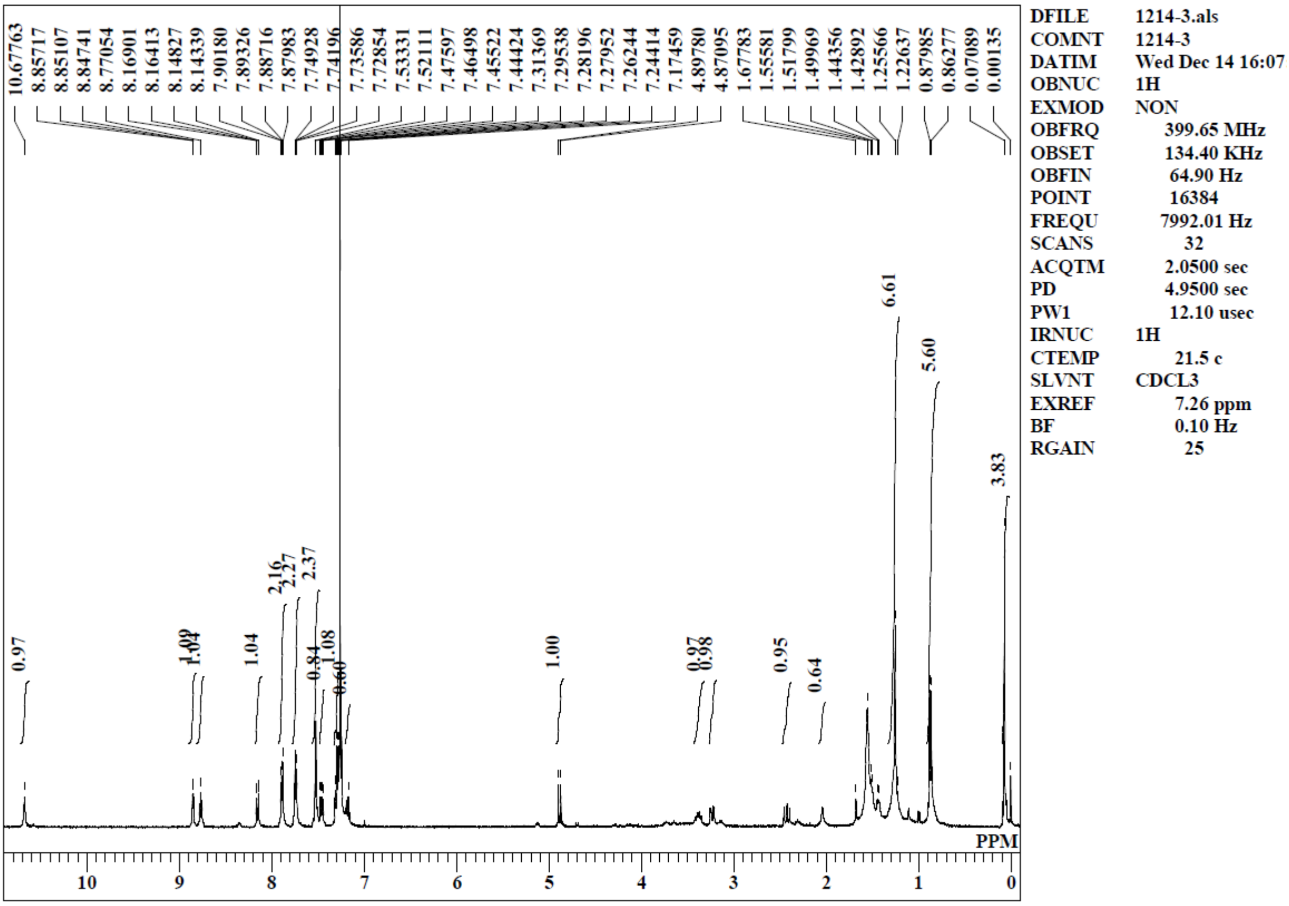

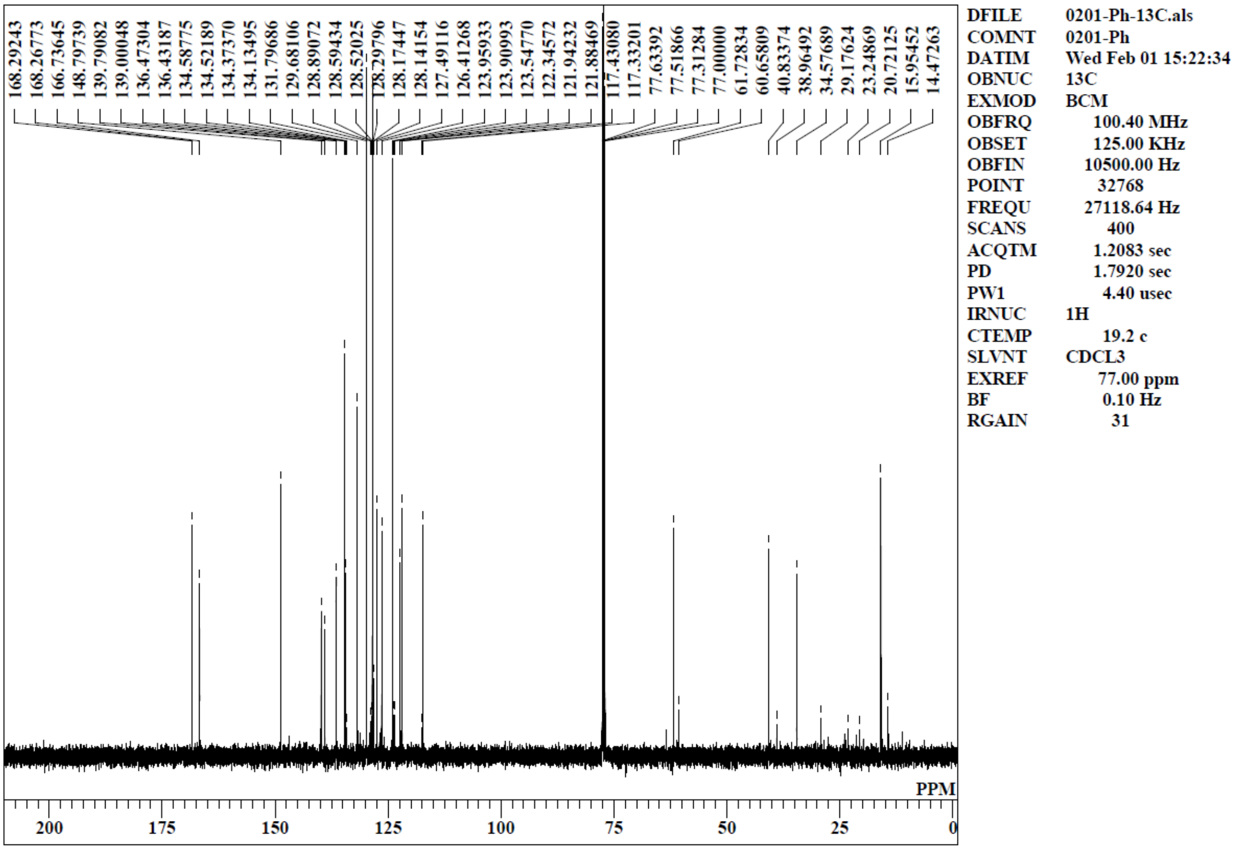

Figure S2. 1H and 13C NMR spectra of compound **6**

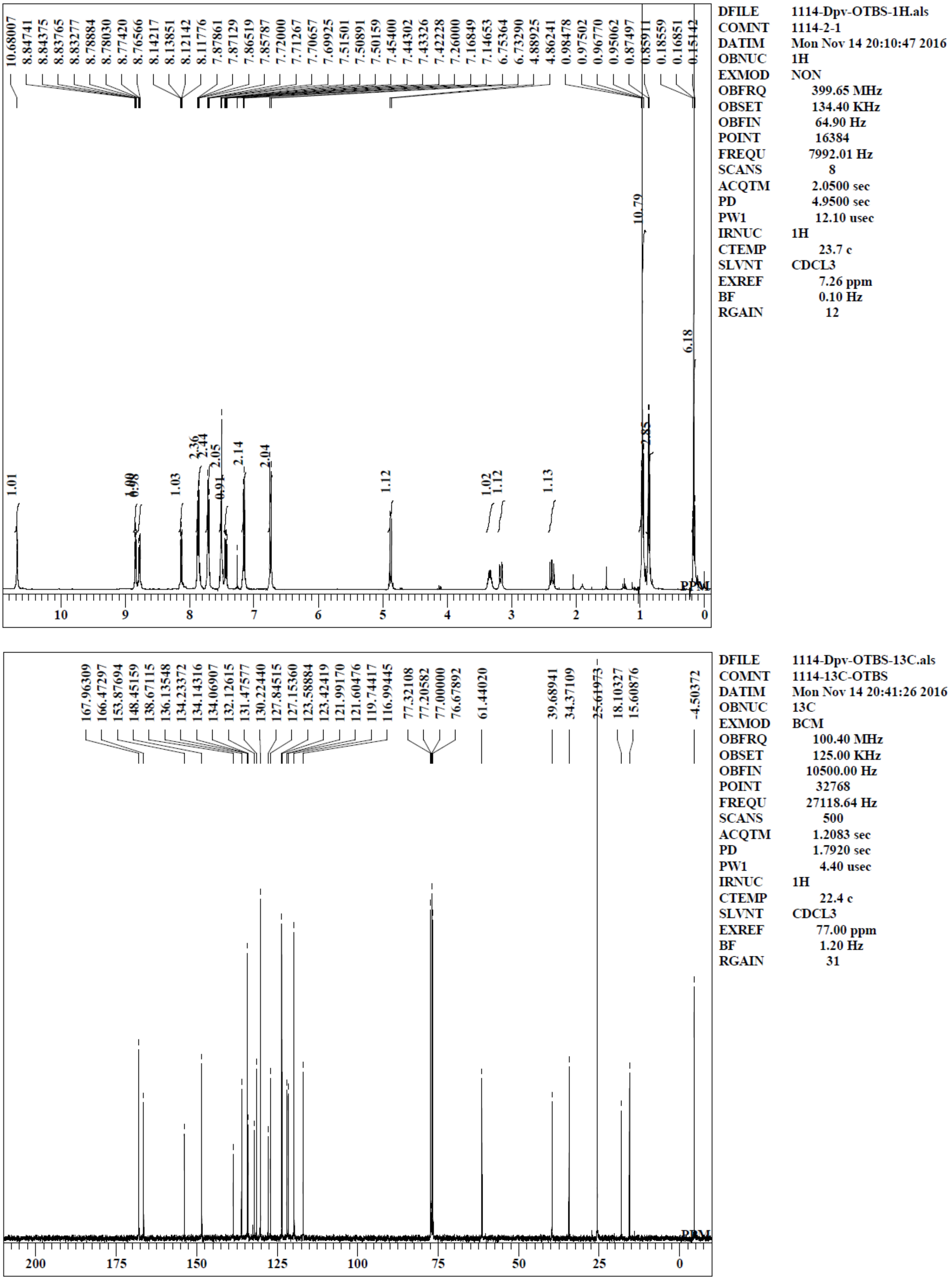

Figure S3. 1H and 13C NMR spectra of compound **8**

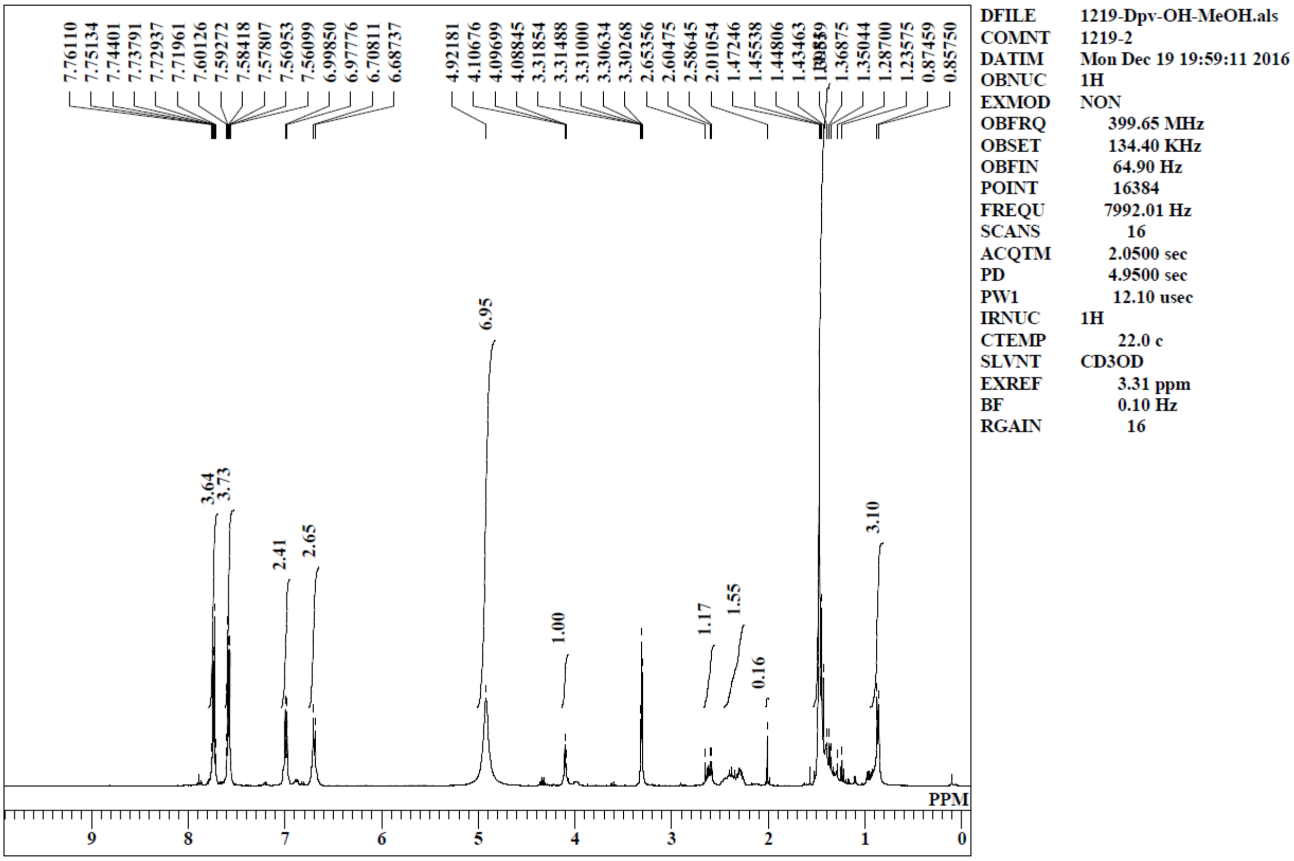

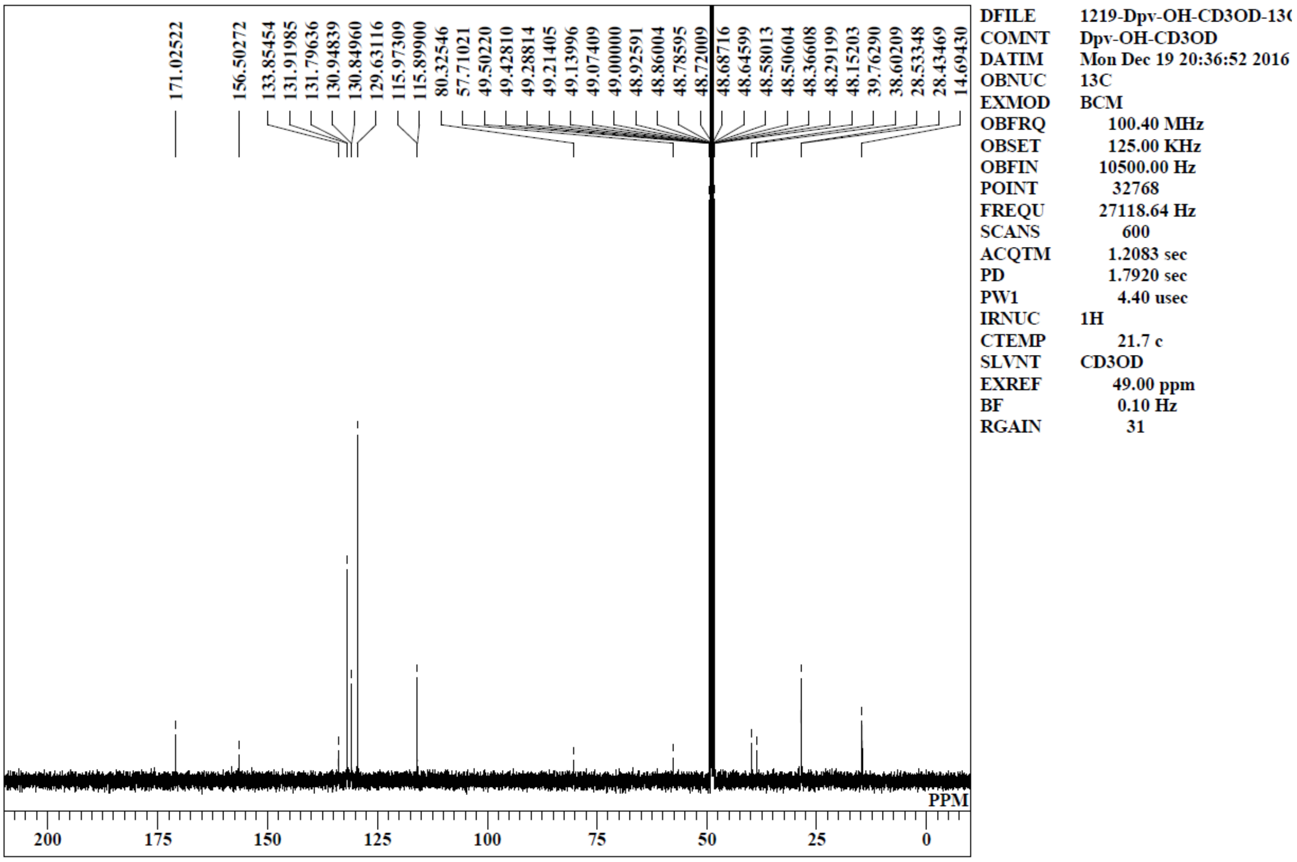

Figure S4. 1H and 13C NMR spectra of compound **13**

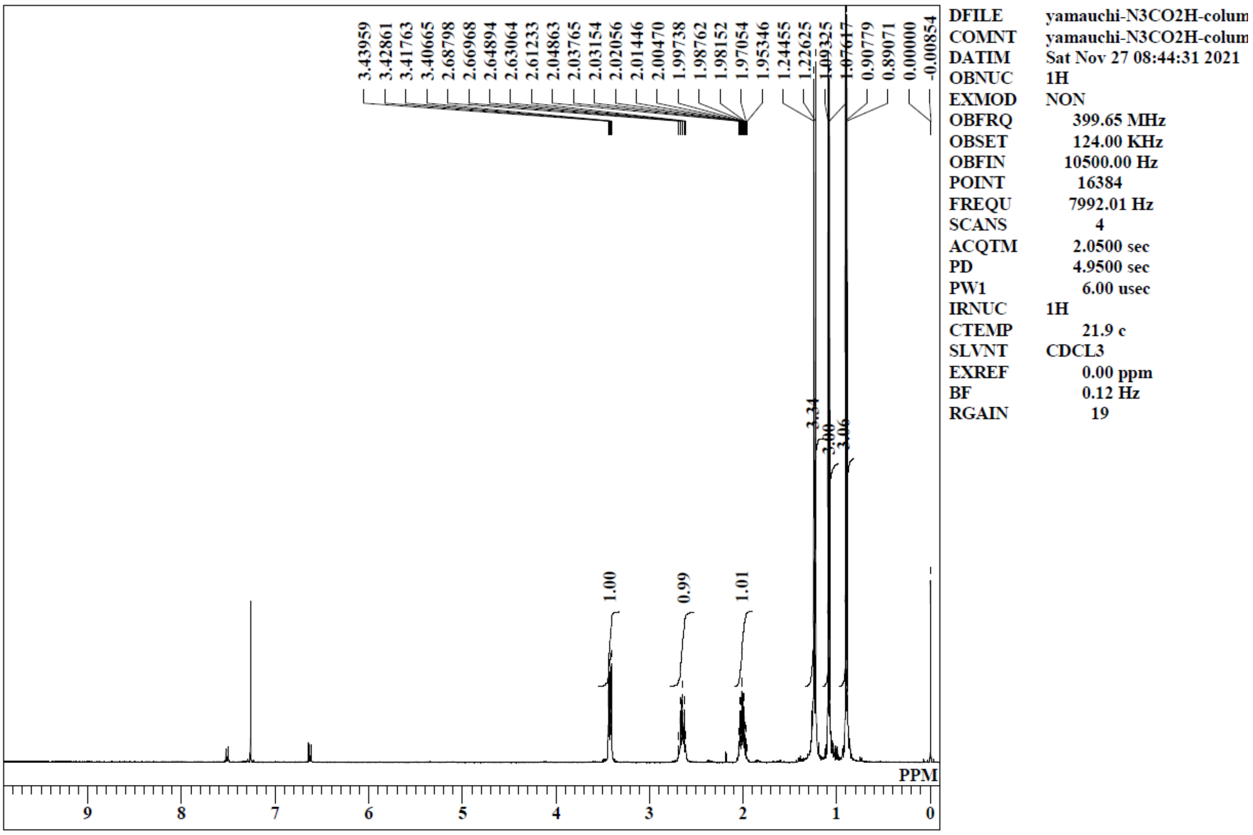

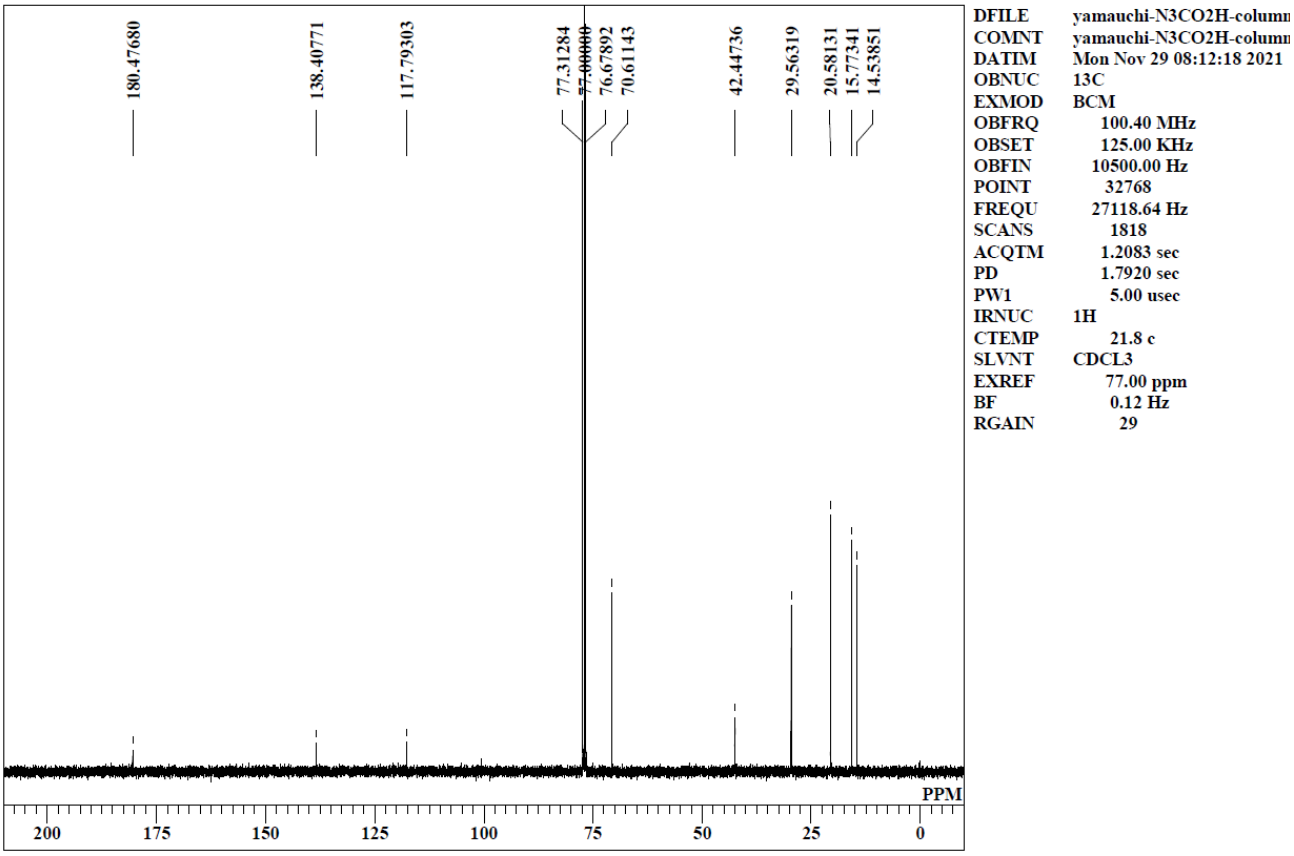

Figure S5. 1H and 13C NMR spectra of compound **14**

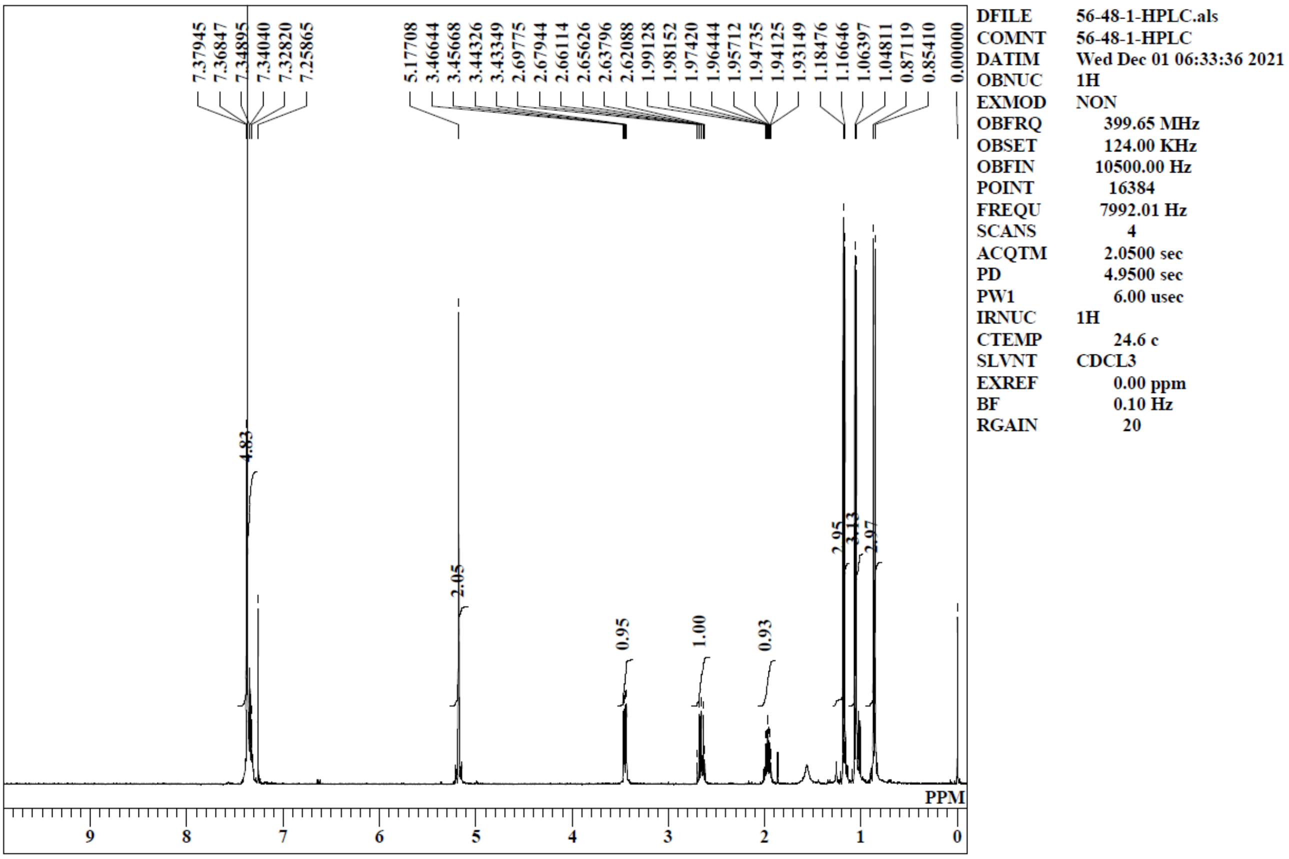

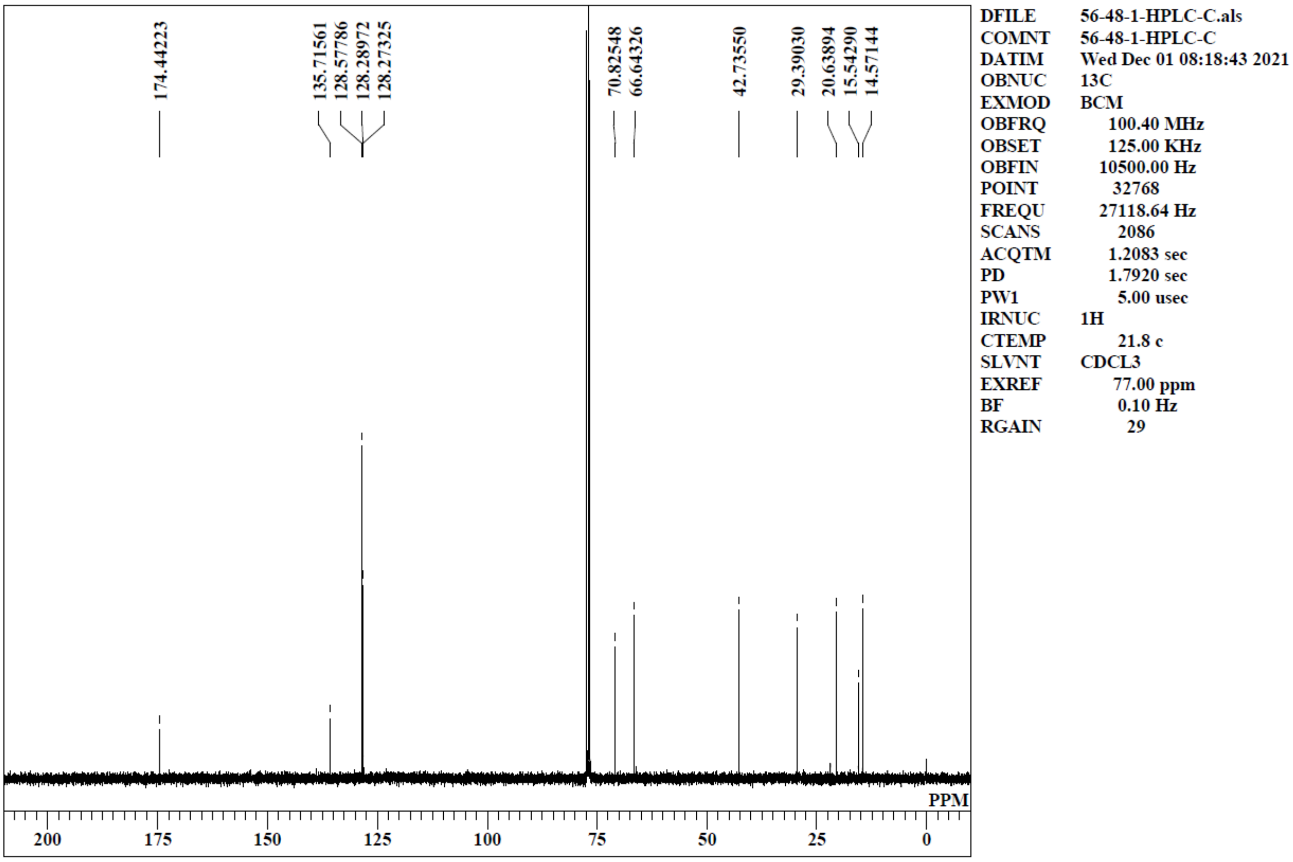

Figure S6. 1H and 13C NMR spectra of compound **16**

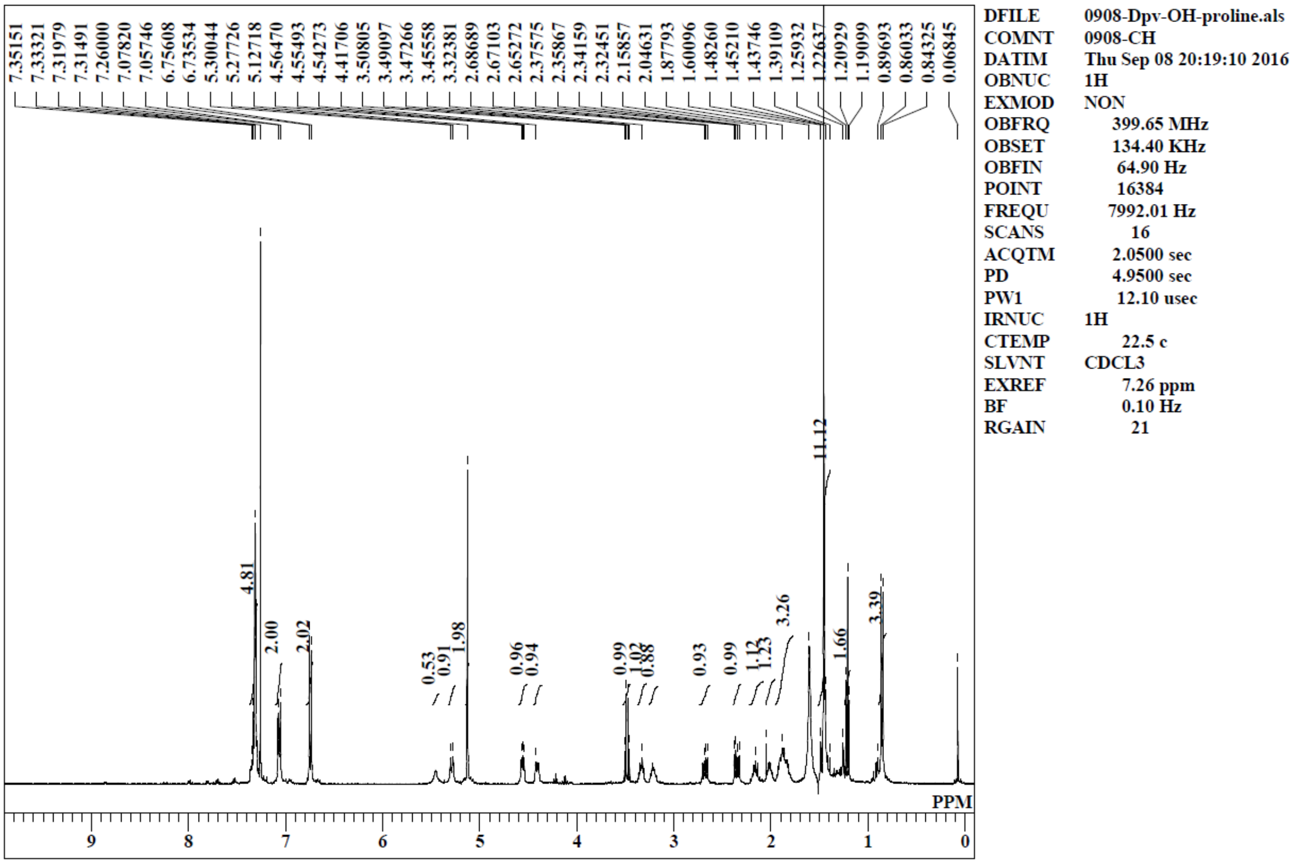

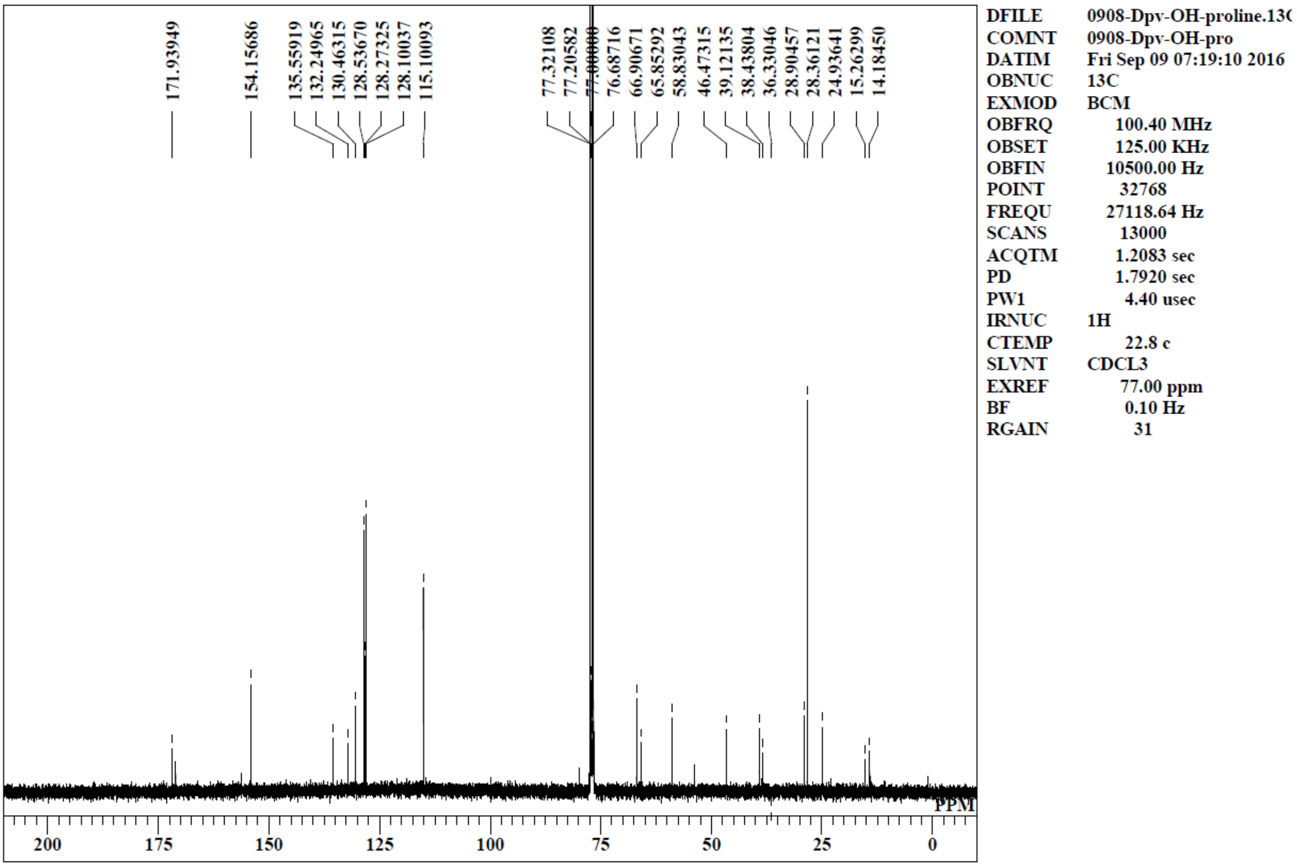

Figure S7. 1H and 13C NMR spectra of compound **17**

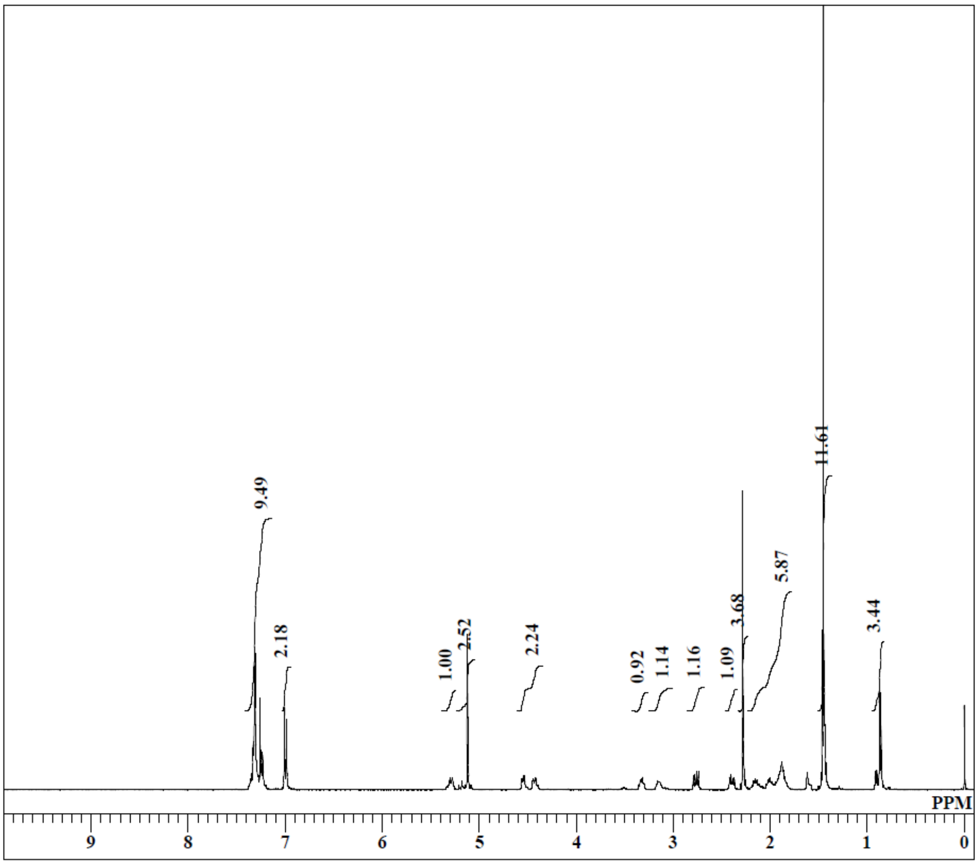

DFILE 2aa-OAc-HPLC1.als
COMNT 2aa-OAc-HPLC
DATIM Thu Nov 25 11:11:51 2021
OBNUC 1H
EXMOD NON
OBFRQ 399.65 MHz
OBSET 124.00 KHz
OBFIN 10500.00 Hz
POINT 16384
FREQU 7992.01 Hz
SCANS 4
ACQTM 2.0500 sec
PD 4.9500 sec
PW1 6.00 usec
IRNUC 1H
CTEMP 23.0 c
SLVNT CDCL3
EXREF 0.00 ppm
BF 0.10 Hz
RGAIN 20

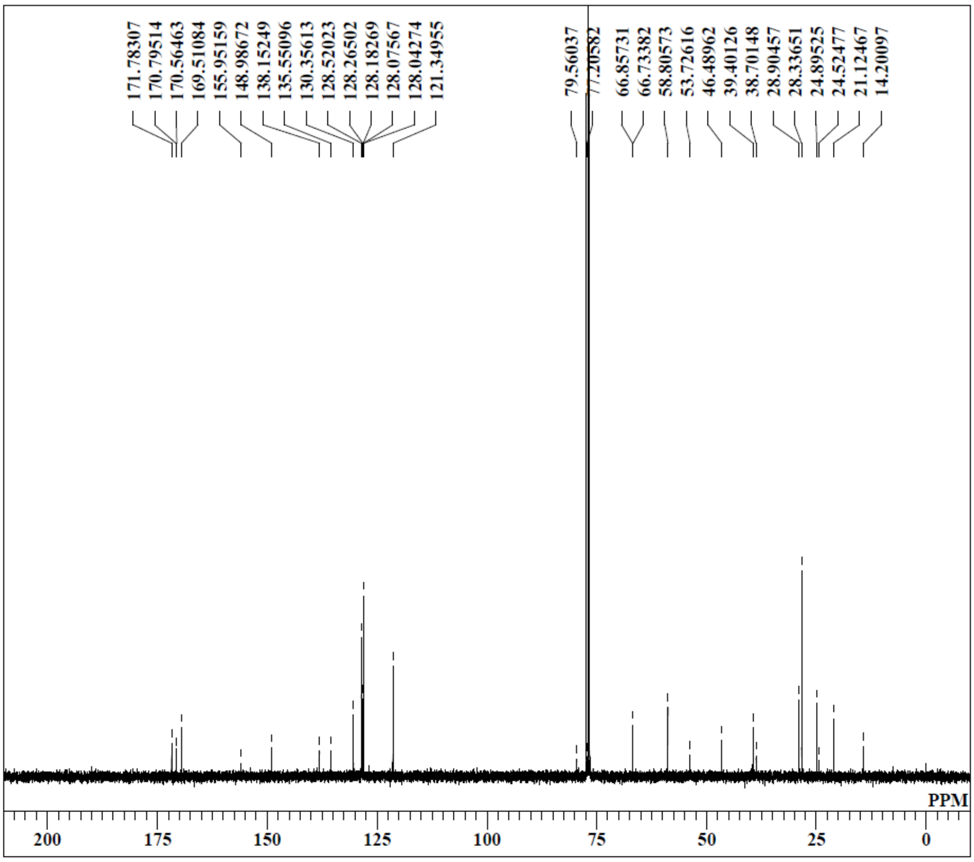

DFILE sou-2aa-OAC-C.als
COMNT sou-2aa-OAC-C
DATIM Sat Nov 27 08:39:21 2021
OBNUC 13C
EXMOD BCM
OBFRQ 100.40 MHz
OBSET 125.00 KHz
OBFIN 10500.00 Hz
POINT 32768
FREQU 27118.64 Hz
SCANS 2002
ACQTM 1.2083 sec
PD 1.7920 sec
PW1 5.00 usec
IRNUC 1H
CTEMP 22.0 c
SLVNT CDCL3
EXREF 77.00 ppm
BF 0.12 Hz
RGAIN 27

Figure S8. 1H and 13C NMR spectra of compound **20**

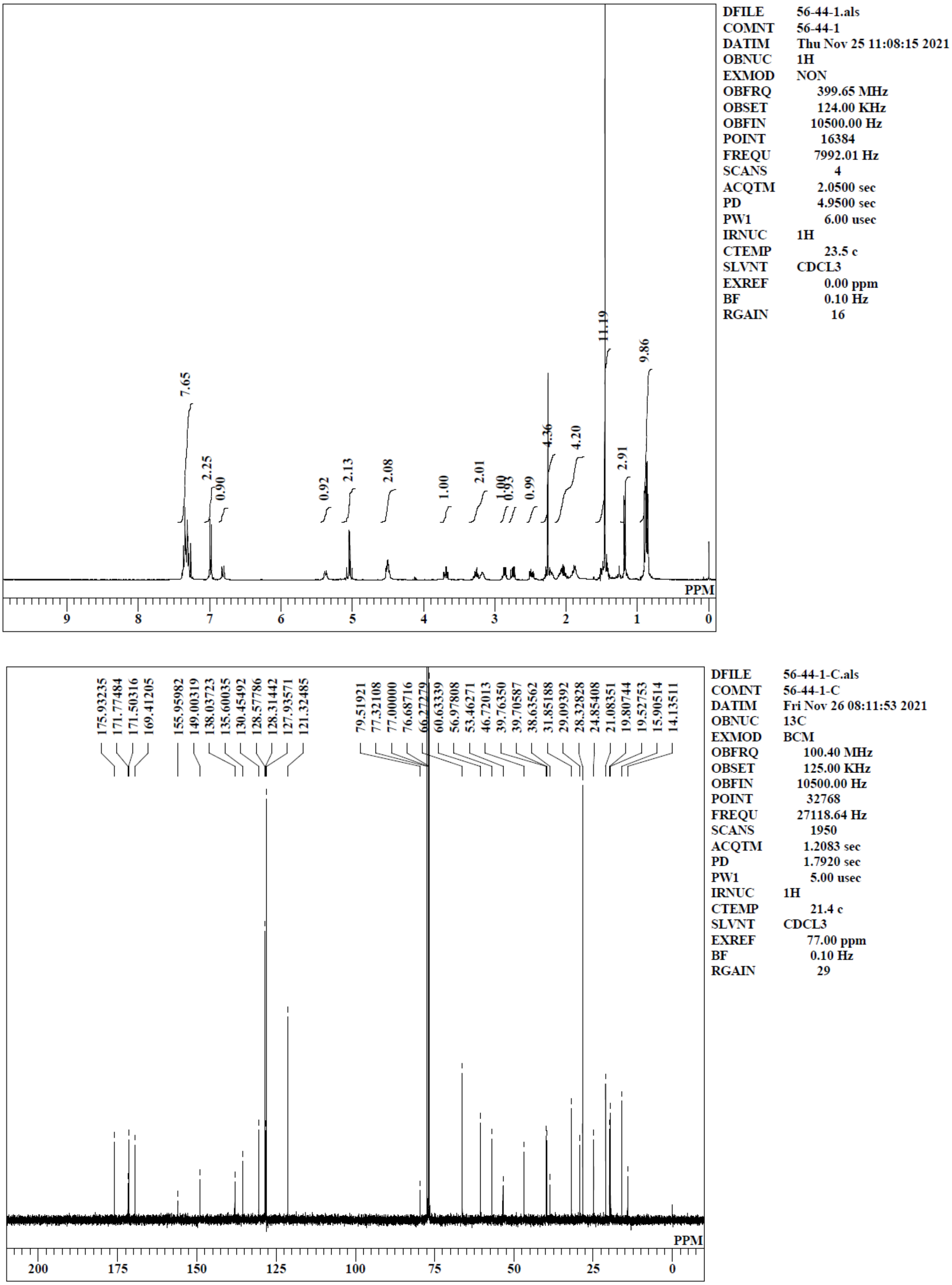

Figure S9. 1H and 13C NMR spectra of compound **21**

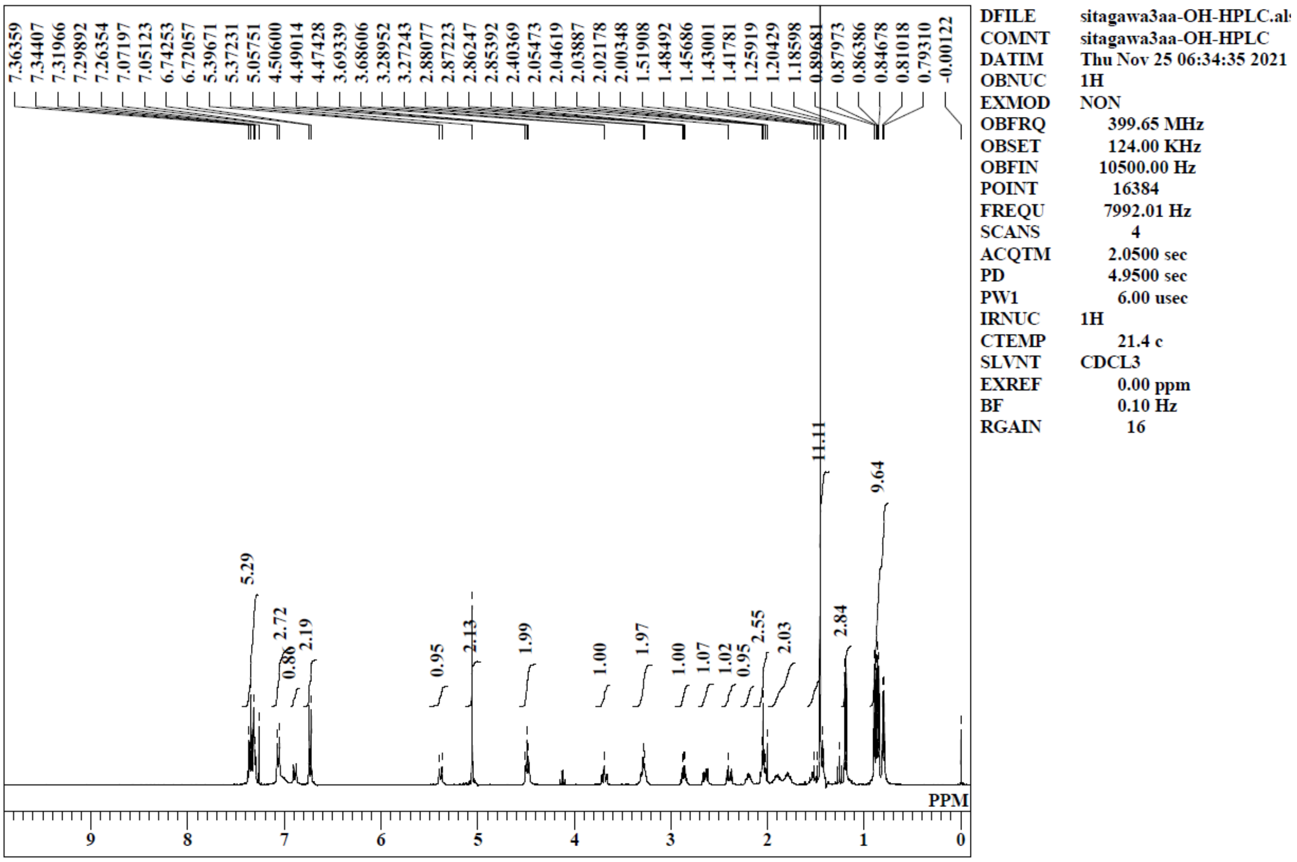

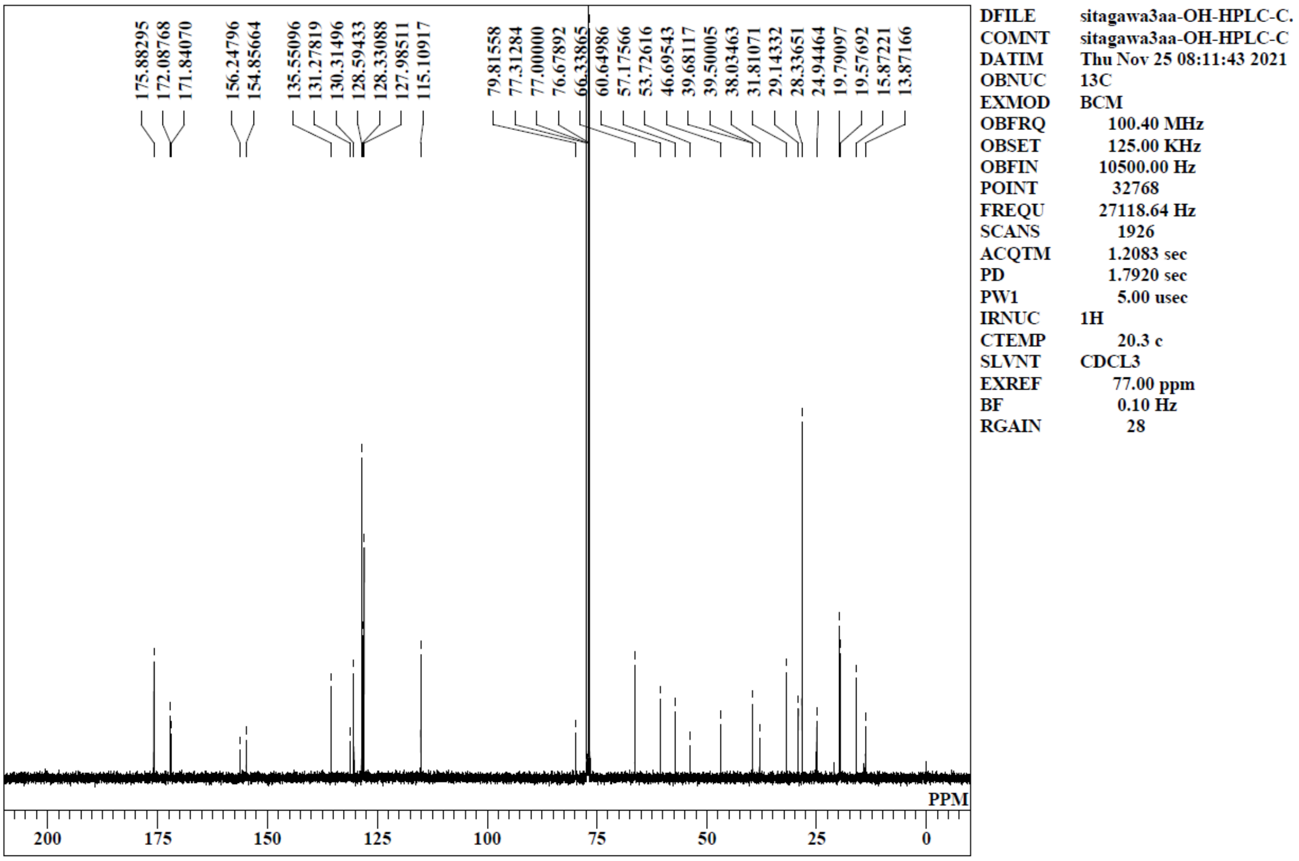

Figure S10. 1H and 13C NMR spectra of compound **22**

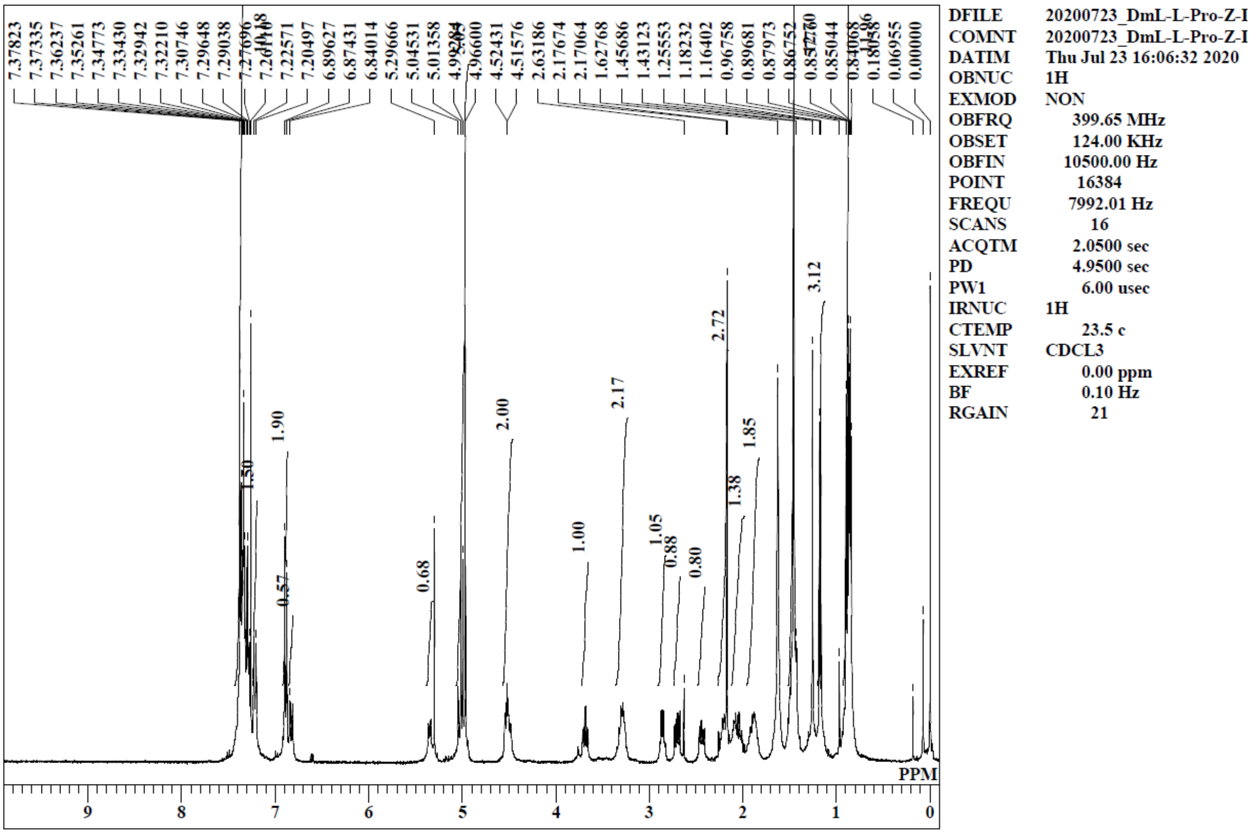

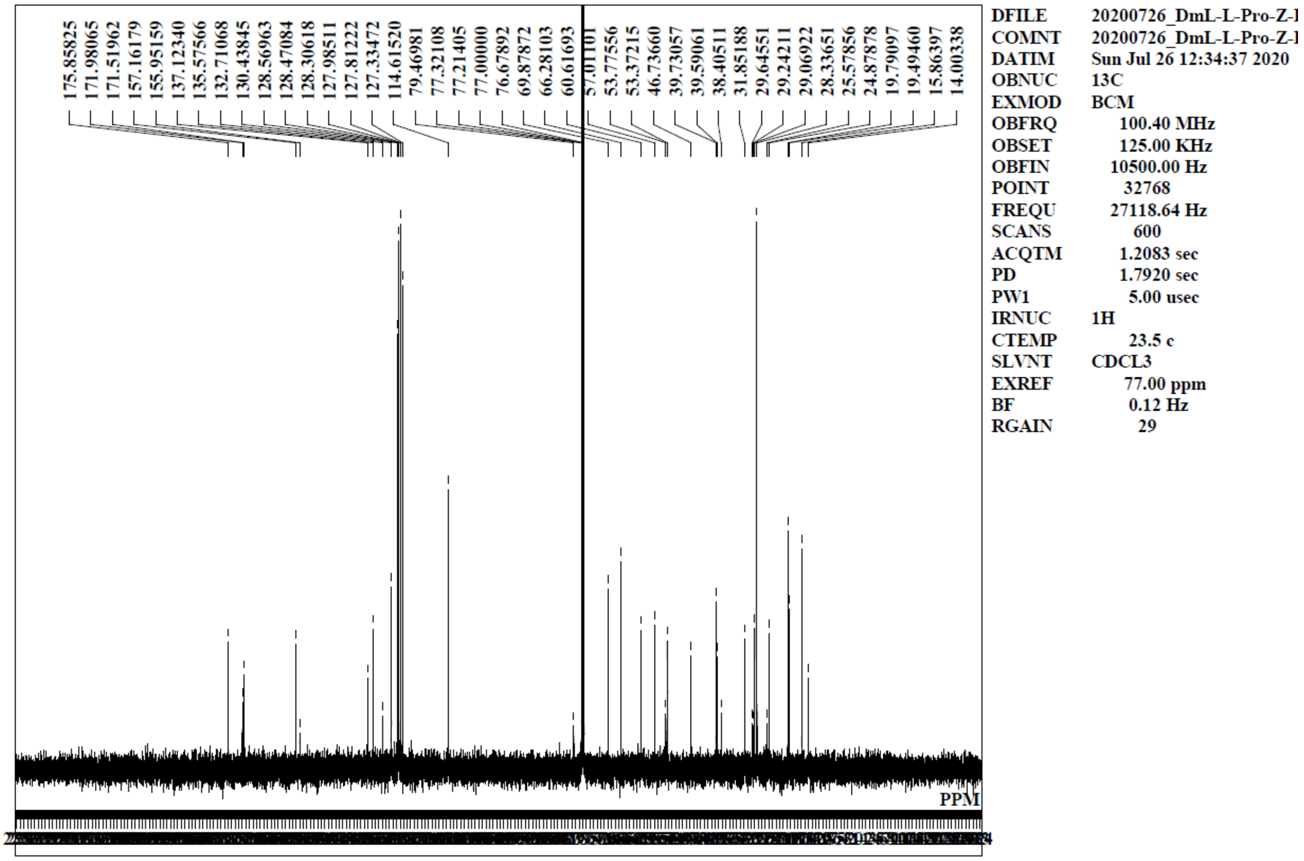

Figure S11. 1H and 13C NMR spectra of compound **23**

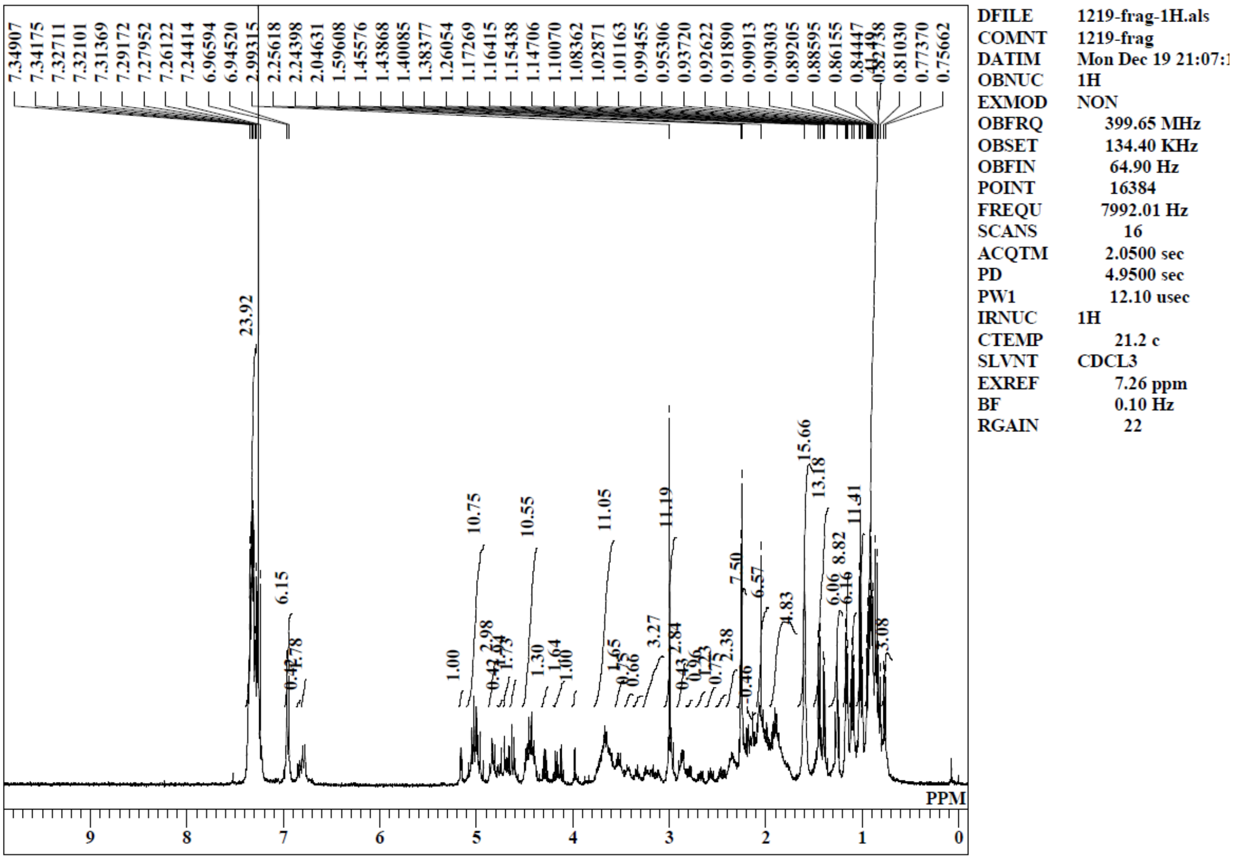

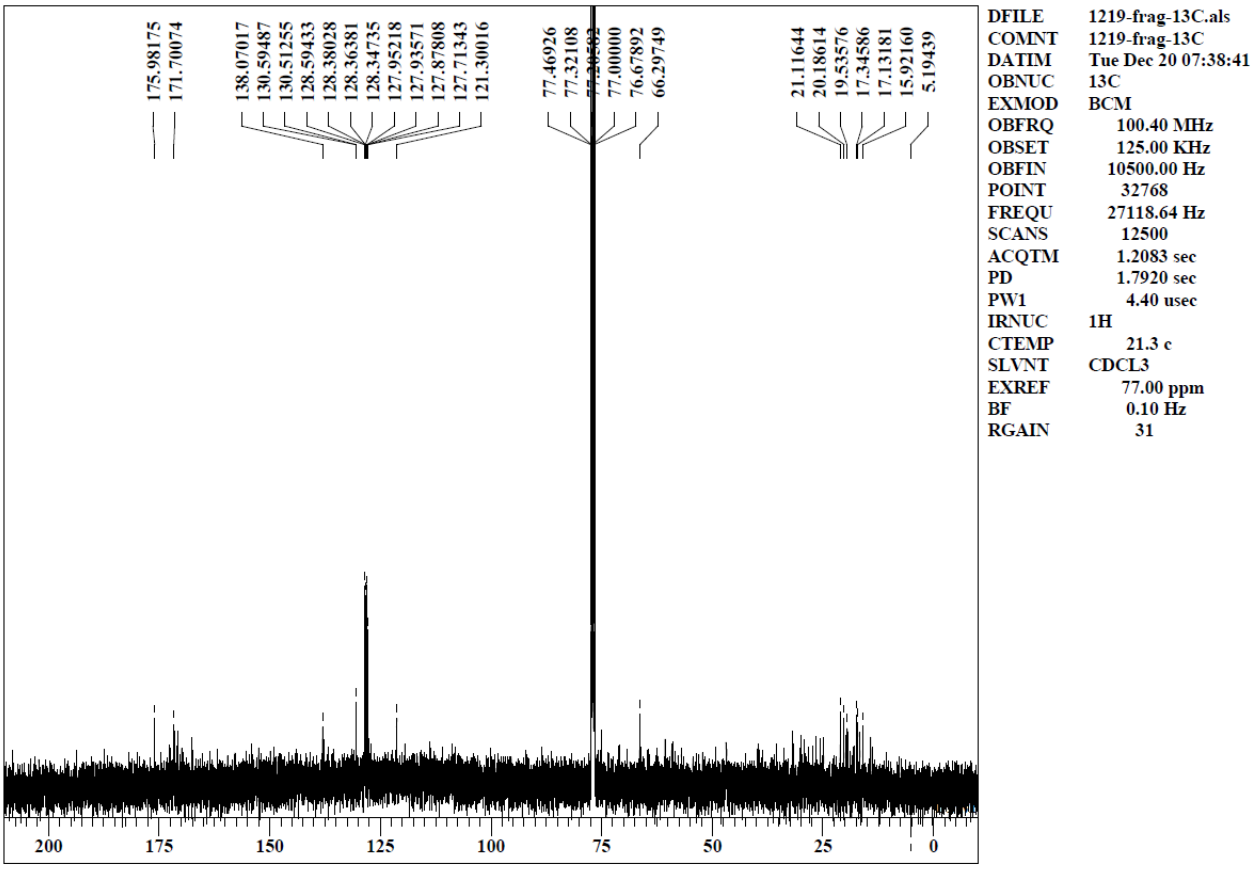

Figure S12. 1H and 13C NMR spectra of compound **24**

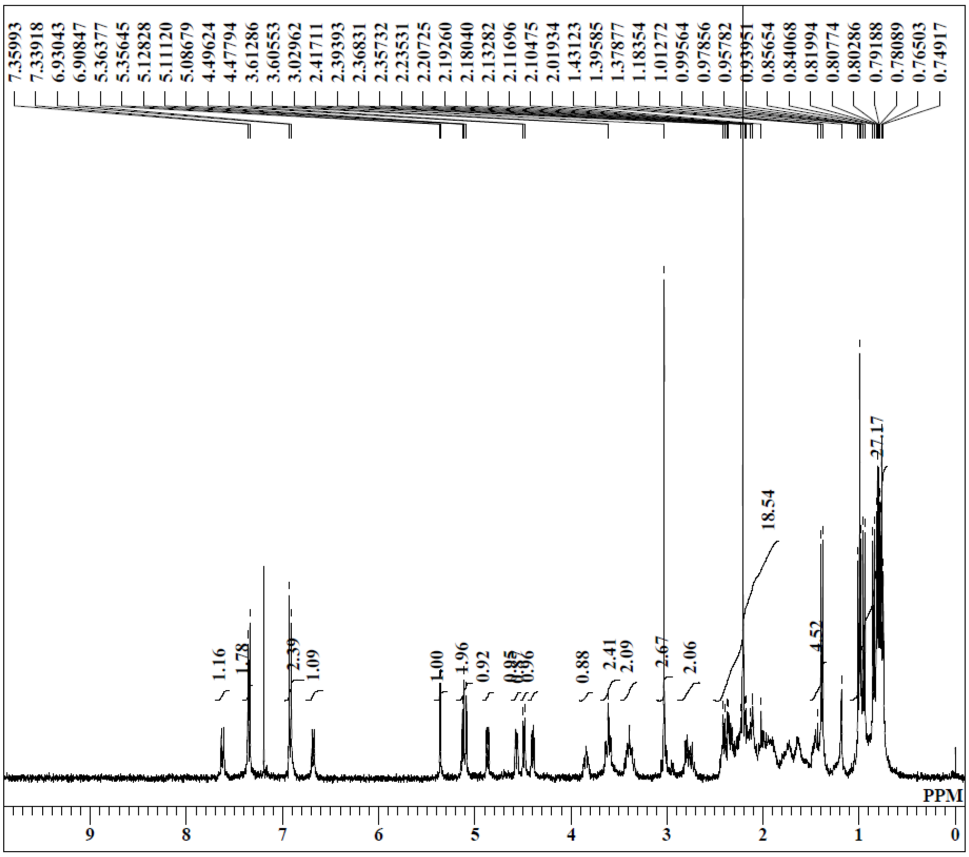

DFILE 56-75-5.als
COMNT 56-75-5
DATIM Mon Dec 27 11:06:23
OBNUC 1H
EXMOD NON
OBFRQ 399.65 MHz
OBSET 124.00 KHz
OBFIN 10500.00 Hz
POINT 16384
FREQU 7992.01 Hz
SCANS 4
ACQTM 2.0500 sec
PD 4.9500 sec
PW1 6.00 usec
IRNUC 1H
CTEMP 20.2 c
SLVNT CDCL3
EXREF 0.00 ppm
BF 0.12 Hz
RGAIN 23

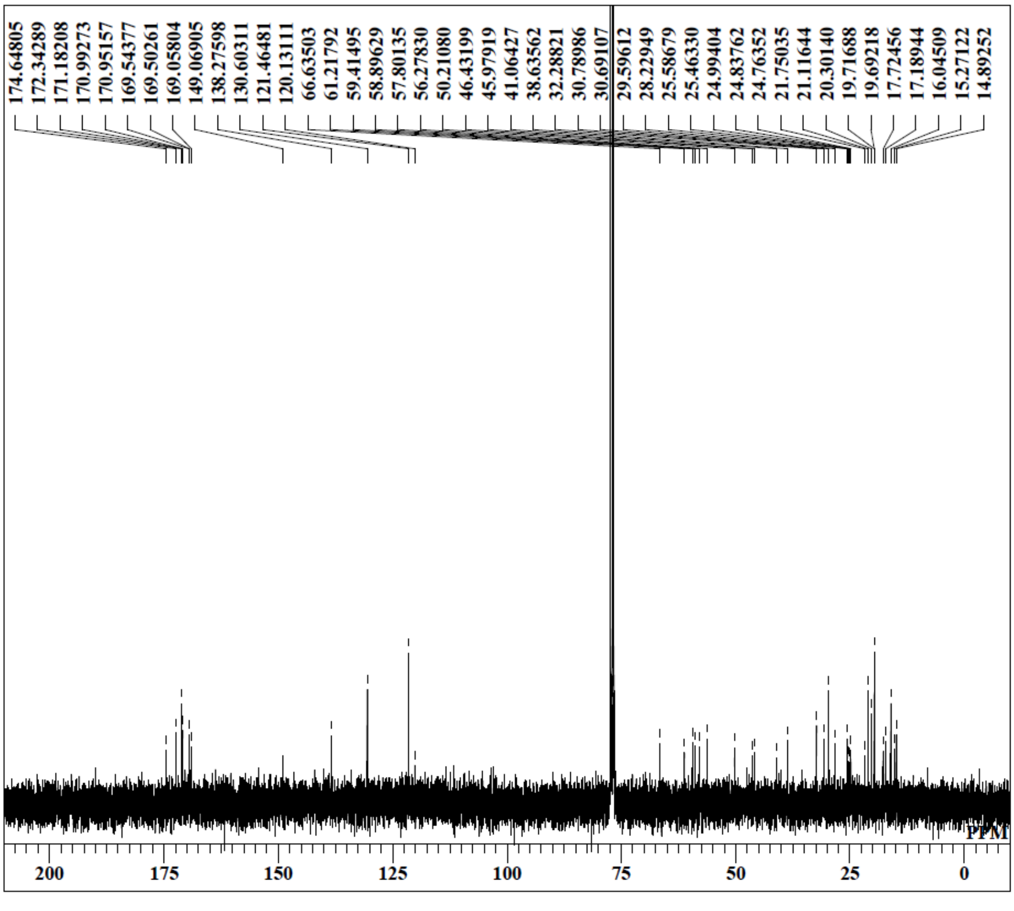

DFILE _DEFAULT.ALS
COMNT 56-75-5-C
DATIM Tue Dec 28 07:04:59
OBNUC 13C
EXMOD BCM
OBFRQ 100.40 MHz
OBSET 125.00 KHz
OBFIN 10500.00 Hz
POINT 32768
FREQU 27118.64 Hz
SCANS 5566
ACQTM 1.2083 sec
PD 1.7920 sec
PW1 5.00 usec
IRNUC 1H
CTEMP 17.6 c
SLVNT CDCL3
EXREF 77.00 ppm
BF 0.12 Hz
RGAIN 28

Figure S13. 1H and 13C NMR spectra of compound **25**

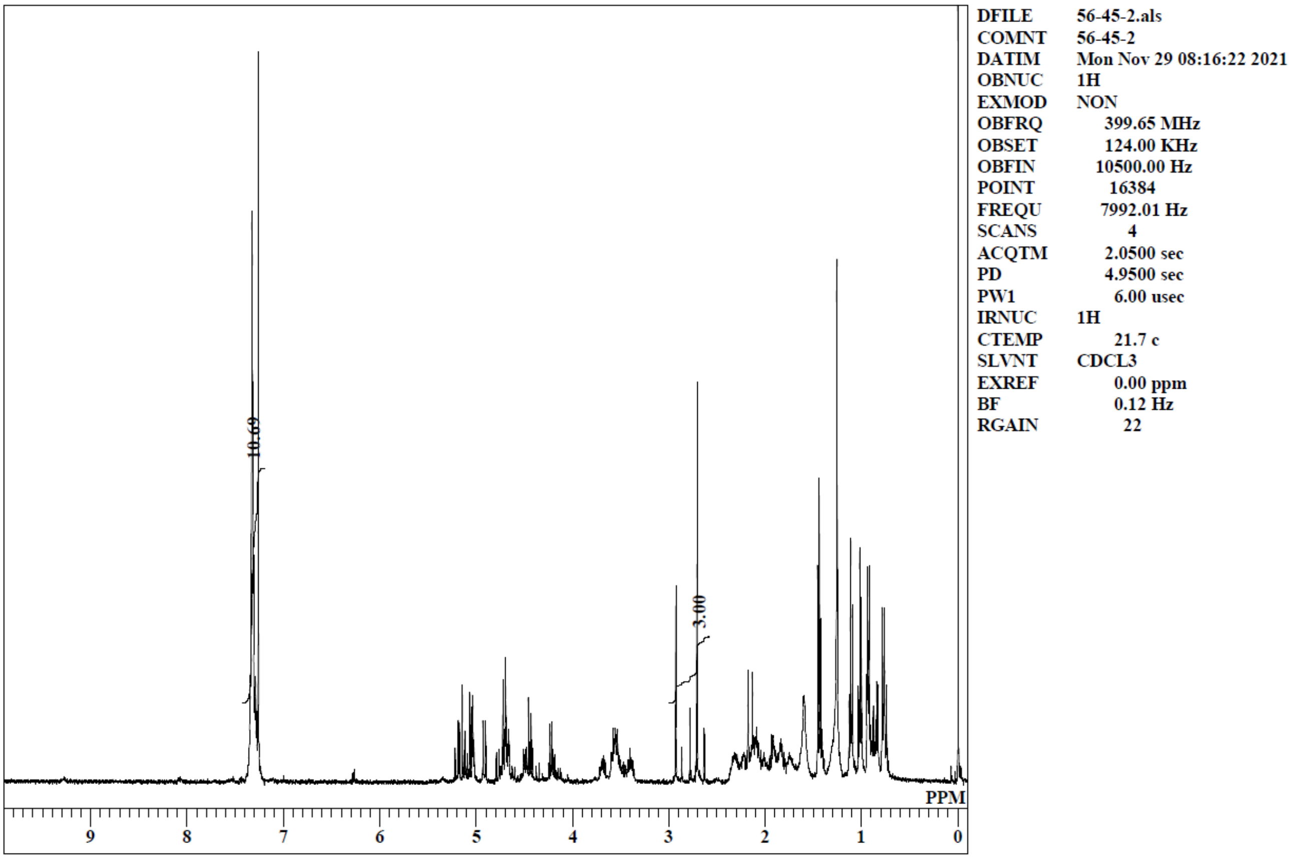

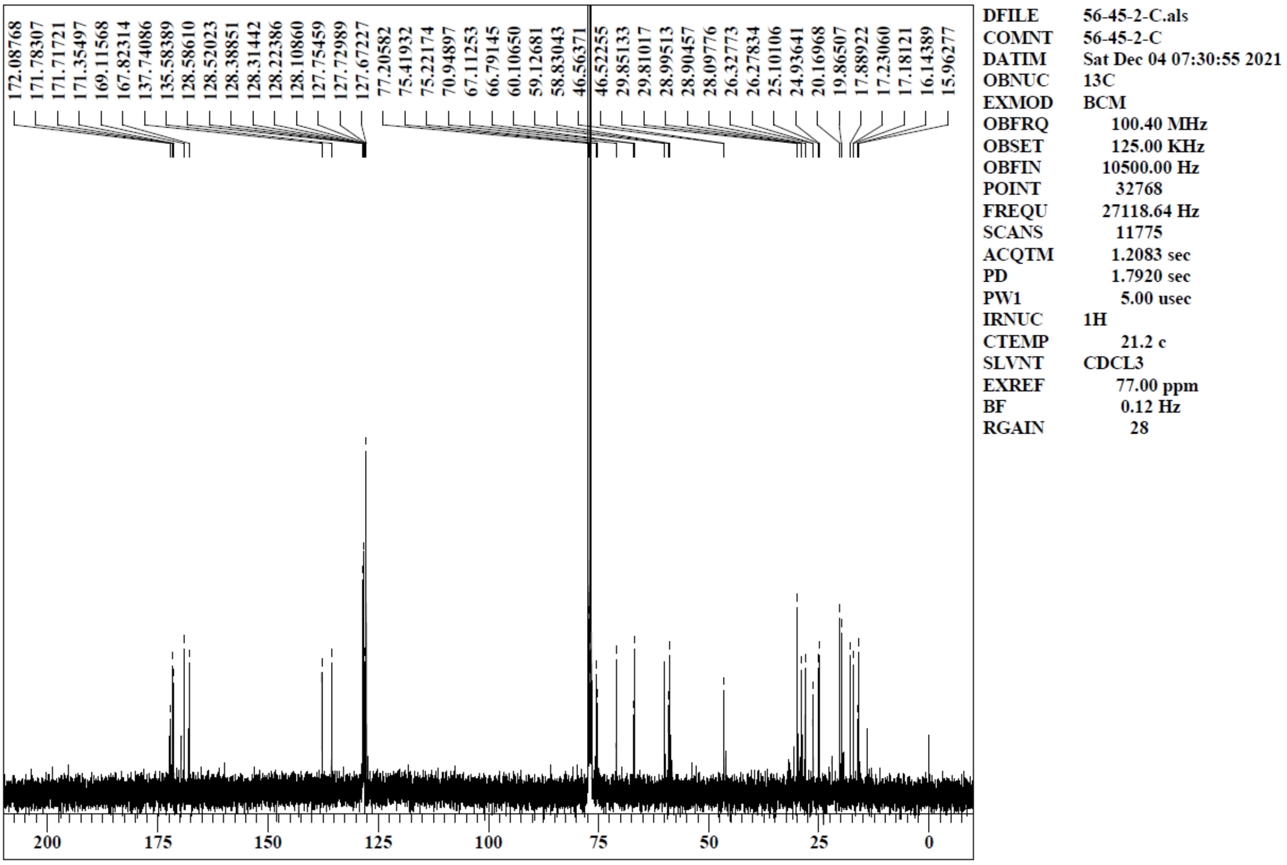

Figure S14. 1H and 13C NMR spectra of compound **29**

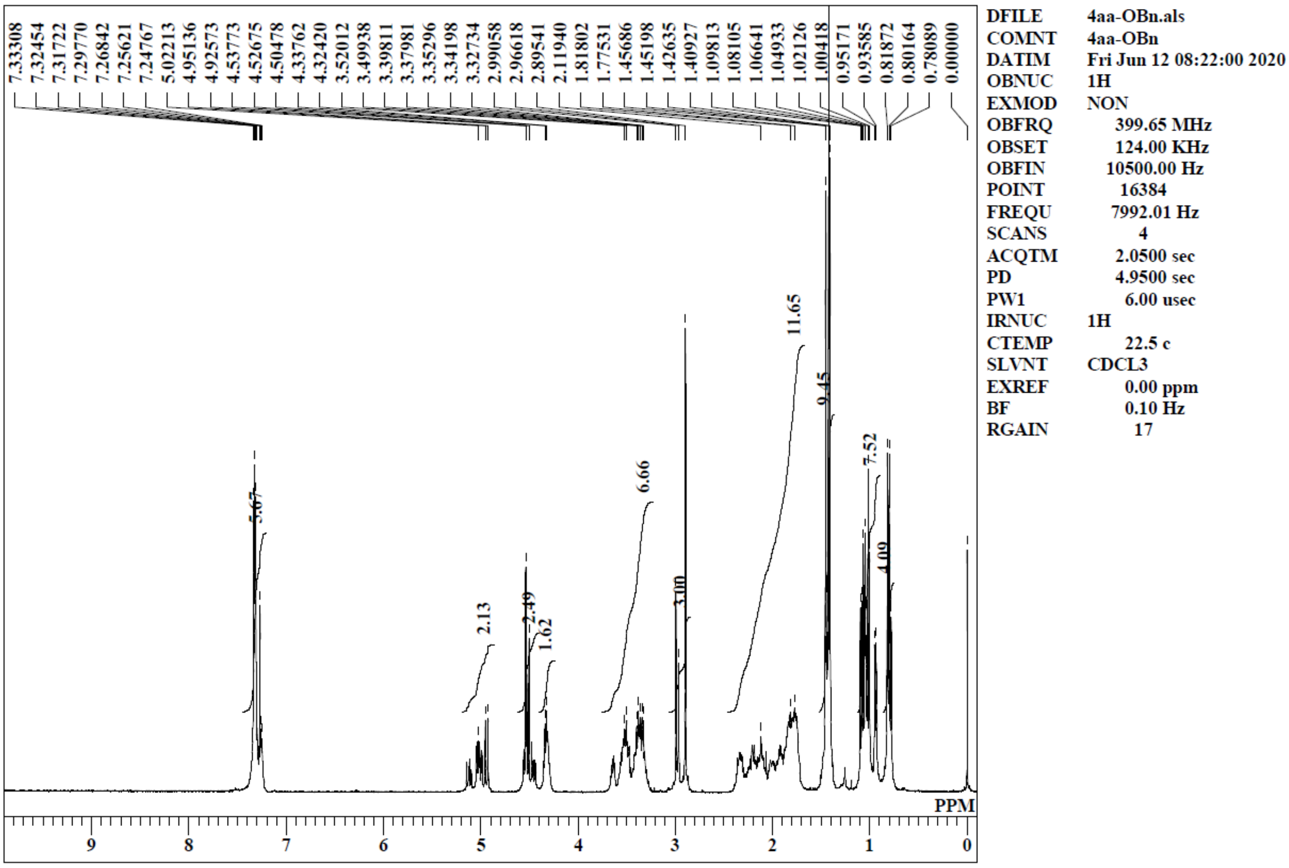

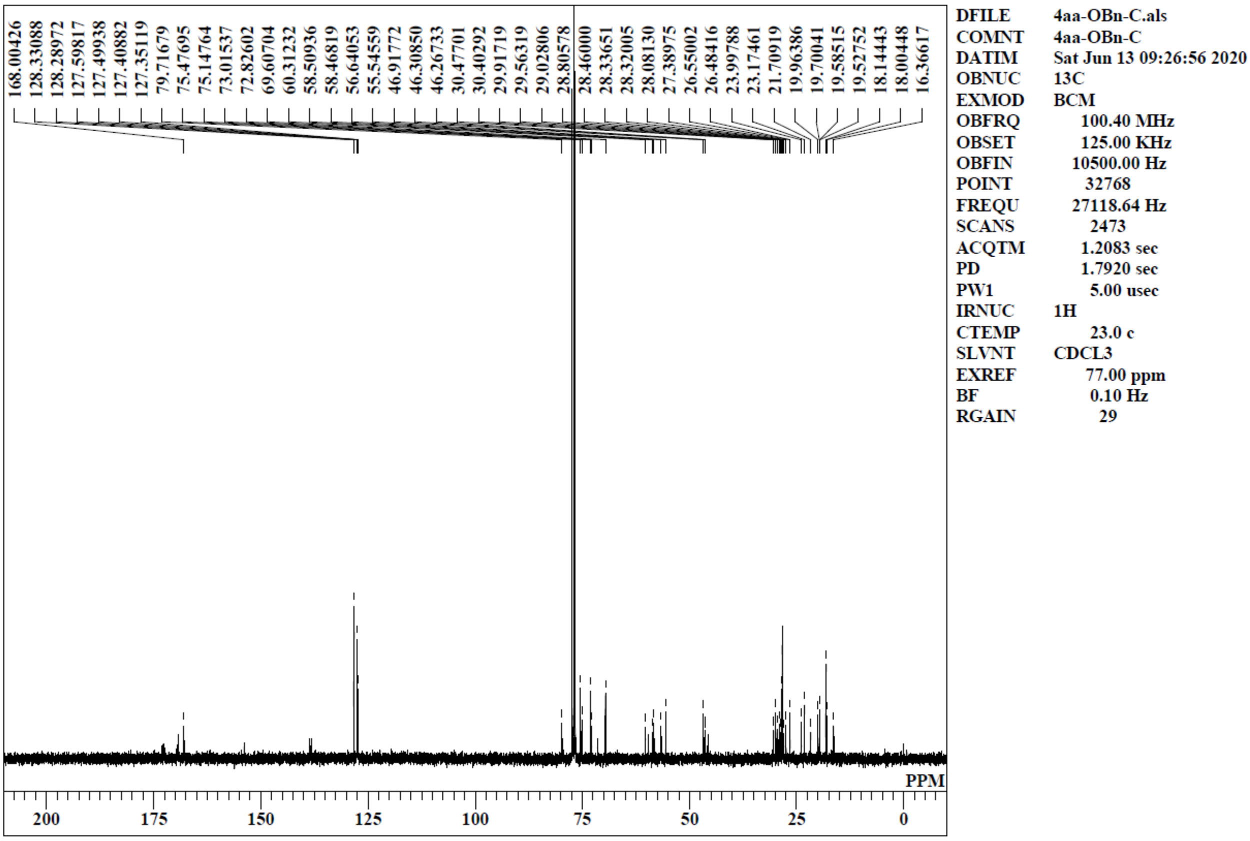

Supplement: Supplementary file 1 [file marinedrugs-20-00124-s001.zip › marinedrugs-1553695-supplementary.pdf]
